# Development of dual base editors based on ssDNA-targeting SCP1.201 deaminases for the artificial evolution of novel herbicide-tolerant *OsEPSPS* variants

**DOI:** 10.1016/j.xplc.2025.101632

**Published:** 2025-12-03

**Authors:** Rongfang Xu, Jiahui Zhu, Hui Wang, Huanhuan Wang, Liangxia Zhao, Xiaoshuang Liu, Ruiying Qin, Lang Pan, Pengcheng Wei, Juan Li

**Affiliations:** 1 Anhui Province Key Laboratory of Rice Germplasm Innovation and Molecular Improvement, Anhui Academy of Agricultural Sciences, Hefei 230031, P.R. China; 2 College of Agronomy, Anhui Agricultural University, Hefei 230036, P.R. China; 3 Research Centre for Biological Breeding Technology, Advanced Academy, Anhui Agricultural University, Hefei 230036, P.R. China; 4 College of Plant Protection, Hunan Agricultural University, Changsha 410128, P.R. China

Dear Editor,

Dual base editors (DuBEs) enable the simultaneous introduction of multiple types of nucleotide substitutions using a single guide RNA (sgRNA), thus greatly broadening the product diversity of base editing. Plant dual cytosine and adenine base editors (A&CBEs) were developed by fusing an apolipoprotein B mRNA-editing enzyme catalytic polypeptide (APOBEC)/activation-induced cytidine deaminase (AID)-like deaminase and an artificially evolved TadA-8e adenine deaminase to the same or opposite termini of a catalytically impaired Cas9 nickase (Li et al., 2020, 2024; Xu et al., 2021; Zhang et al., 2023a, 2023b; Zheng et al., 2024). A bifunctional TadDE derived from TadA-8e has also been used for dual C-to-T and A-to-G editing in rice (Fan et al., 2024; Yu et al., 2024). Taking advantage of these DuBEs, *in situ* targeted saturation mutagenesis and directed artificial evolution have been performed in crops to improve breeding traits (Li et al., 2024). Recently, numerous novel cytidine deaminases were identified through an AI-assisted 3D protein structure–based clustering strategy (Huang et al., 2023). Among them, a number of single-stranded DNA (ssDNA)-targeting proteins from the SCP1.201 clade (Sdds) have been engineered as compact CBEs, enabling efficient C-to-T editing at conventionally uneditable targets in crop genomes (Huang et al., 2023). However, the dual-base-editing compatibility of Sdds has not yet been assessed.

To investigate appropriate architectures for Sdd-based DuBEs, Sdd6 and Sdd7, the two most well-used and highly promising candidates (Huang et al., 2023), were separately fused to the C or N terminus of the SpCas9 (D10A) nickase (Supplemental Figure 1). The efficiency of the resulting Sdd-CBEminis was assessed at four targets (*DL*-T, *IPA1*-T1, *LAZY1*-T, and *SLR1*-T1) by amplicon next-generation sequencing of stably transformed rice calli (supplemental methods). The average cytosine conversion efficiencies of Sdd7c-CBEmini (Sdd7 fused to the C terminus of nSpCas9) and Sdd7n-CBEmini (Sdd7 fused to the N terminus) were 42.70% and 49.89%, respectively, and they outperformed their Sdd6 counterparts by 7.94- and 2.64-fold (Figure 1A). The Sdd7-CBEminis also produced more C-to-T and C-to-G edits than the Sdd6-derived editors (Supplemental Figure 2). Intriguingly, it appeared that the efficiency of the Sdd6-derived editors depended on the specific terminus to which Sdd6 was fused. The efficiency of Sdd6n-CBEmini was 2.44- to 7.08-fold higher than that of Sdd6c-CBEmini at all four targets (Supplemental Figure 3, *P* < 0.05), suggesting that Sdd6 was more effective as an N-terminal fusion. By contrast, Sdd7n-CBEmini and Sdd7c-CBEmini exhibited similar efficiencies at the *DL*-T, *SLR1*-T1, and *LAZY1*-T1 sites (*P* > 0.05), although the efficiency of Sdd7n-CBEmini was slightly higher (1.39-fold) than that of Sdd7c-CBEmini at the *IPA1*-T1 site (Supplemental Figure 3). In addition, Sdd7c- and Sdd7n-CBEmini exhibited the same editing range between positions 3 and 12 (counting from the distal end of the protospacer; Supplemental Figure 4). These results suggest that Sdd7 does not have an intrinsic terminus preference for base editing. 3D structure predictions revealed that, unlike those of Sdd6n-CBEmini, Sdd7c-CBEmini, and Sdd7n-CBEmini, the N-terminal region and α-helical domain of Sdd6 in Sdd6c-CBEmini were largely disordered and exhibited significant spatial separation from Cas9, inducing potential steric hindrance that may impair editing activity (Supplemental Figure 5).

**Figure 1:**
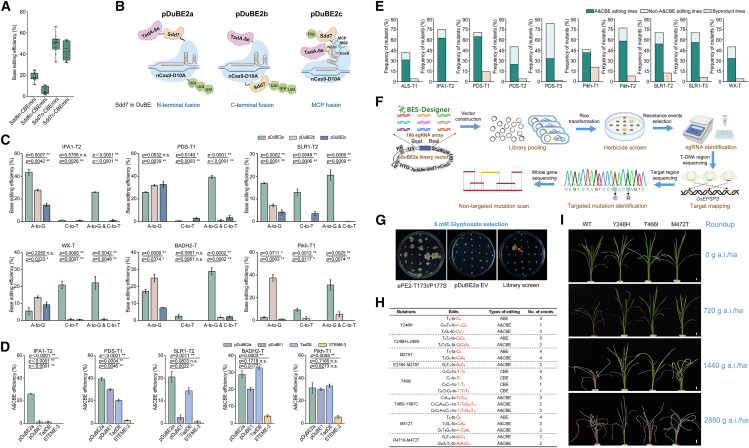
Editing and applications of Sdd-derived dual base editors in rice. **(A)** Base-editing efficiencies of Sdd6- and Sdd7-derived CBEminis. Efficiency was expressed as the ratio of the cytosine-edited reads to total reads. Boxplots show the editing at four endogenous targets with three biological replicates. **(B)** Diagram of Sdd7 dual base editors. pDuBE2a, both Sdd7 and TadA-8e fused to the N-terminus of an SpCas9 D10A nickase (Cas9-D10A), followed by C-terminal UGIs; pDuBE2b, Sdd7 and TadA-8e fused to the opposite terminus of Cas9-D10A; pDuBE2c, MS2, and boxB integrated into the sgRNA scaffold to recruit MCP-Sdd7-UGI and N22p-TadA-8e, respectively. **(C)** Base-editing efficiency of pDuBE2s in rice calli. A-to-G, C-to-T, and concurrent A-to-G and C-to-T conversions are defined as ABE, CBE, and A&CBE outcomes, respectively, of the dual base editors. Mean efficiencies and standard deviations are presented (*n* = 3). *P* values were determined by one-way ANOVA. ∗∗*P* < 0.01, ∗*P* < 0.05, and n.s., not significant. **(D)** Editing efficiencies of the plant dual base editors pDuBE2a, pDuBE1, TadDE, and STEME-3 were quantified by amplicon sequencing. The A&CBE editing efficiency was calculated as the percentage of sequencing reads with concurrent A-to-G and C-to-T substitutions relative to the total number of clean reads. Values shown are the mean ± standard deviation from three biological replicates across five independent genomic target sites. **(E)** Base editing of pDuBE2a in T_0_ transgenic rice plants. The ratios of edited lines are presented. To ensure heritability, a threshold of 15% in the Hi-TOM (high-throughput tracking of mutations) assay was used to filter the edits. Lines with A&CBE edits (simultaneous A-to-G and C-to-T editing in one allele) are shown in dark turquoise, and lines with only individual A-to-G and/or C-to-T edits are shown in light turquoise. Plants with byproducts are shown in light peach. **(F)** Schematic illustration of the procedure for evolving glyphosate-resistant *OsEPSPS* mutants with the pDuBE2a library. A simplified array of 160 sgRNAs was designed for saturation mutagenesis of *OsEPSPS* with the BES-Designer tool. The array was assembled into pDuBE2a to pool a dual-editing library for rice transformation. The T-DNA regions of glyphosate-tolerant callus events were genotyped, and the mutations were identified. The complete *OsEPSPS* gene was then scanned to identify any unintended mutations in the remaining sequence by potential bystander editing or transient expression of unintegrated sgRNA. **(G)** Representative screen of glyphosate-resistant calli. Transformation with the ePE2 (prime editing) vector to induce a T173I-P177S mutation in *OsEPSPS* was used as a positive control for glyphosate selection, and transformation with the pDuBE2a empty vector (EV) was used as a negative control. Calli pooled with the pDuBE2a library were screened by 6 mM glyphosate selection for 4–6 weeks, and a representative plate is shown. The red arrow highlights a resistant callus. **(H)** Glyphosate-resistant *OsEPSPS* mutants screened by pDuBE2a with the 160-sgRNA array. From left to right, mutated residues identified in resistant calli; base edits of *OsEPSPS* in resistant calli: missense mutations resulting in amino acid changes are highlighted in red, synonymous substitutions of adjacent residues are shown in green, and the numbers indicate the positions of the edited nucleotides within the target sequences; types of pDuBE2a activity that produced the edits; and numbers of callus events harboring the mutations. Some events may be chimeras of different variants. **(I)** Herbicide resistance of library-screened mutations in the T_1_ generation. The mutants were reproduced by prime editing to ensure precise mutations. Three-week-old T_1_ heterozygous lines of T466I and T_1_ homozygous lines of Y248H or M472T (as described in Supplemental Table 2) were sprayed with serial dilutions of the commercial herbicide Roundup. The plants were imaged after 2 weeks of incubation. Scale bar, 1 cm.

Because the Sdd7-CBEminis exhibited better cytosine editing efficiency in the rice genome, Sdd7 was chosen for integration with TadA-8e at the same or opposite terminus of nSpCas9. The TadA-8e–Sdd7–nSpCas9–3×UGI and TadA-8e–nSpCas9–Sdd7–3×UGI fusions were designated plant DuBE2a (pDuBE2a) and pDuBE2b, respectively (Figure 1B; Supplemental Figure 6). A multiplex orthogonal base-editor architecture consisting of the nSpCas9 (D10A), MCP–Sdd7–3×UGI, TadA-8e–N22p, and sgRNA–boxB–MS2 modules was also established and designated pDuBE2c. To characterize the editing profiles of the pDuBE2s, six sgRNAs targeting multiple As and Cs were designed. Amplicon next-generation sequencing revealed that pDuBE2a, pDuBE2b, and pDuBE2c produced targeted mutations in 58.02%, 27.08%, and 15.33% of reads, respectively, most of which were C-to-T and/or A-to-G conversions (Supplemental Figure 7). The overall base-editing efficiency of pDuBE2a was significantly higher than that of pDuBE2b at five of the six sites (excluding *Pikh*-T1) by 1.81- to 7.06-fold and higher than that of pDuBE2c at all targets by 1.67- to 41.86-fold (*P* < 0.05, Figure 1C). In terms of pDuBE2a outcomes, edits carrying C-to-T conversions accounted for 27.43% to 43.06% of the reads, much higher than the 0%–7.67% of pDuBE2b and the 0%–6.78% of pDuBE2c (*P* < 0.05, Supplemental Figure 8). The average efficiency of A-to-G conversions produced by pDuBE2a was 46.78%, 1.84- and 3.62-fold higher than the efficiencies of pDuBE2b and pDuBE2c, respectively (*P* < 0.05). The dual A-to-G and C-to-T editing efficiency of pDuBE2a ranged from 20.58% to 39.39% and was significantly higher than that of pDuBE2b and pDuBE2c by at least 5.62-fold (Figure 1C, *P* < 0.05). In contrast to the low proportions of A&CBE products generated by pDuBE2b (0%–11.83%) and pDuBE2c (0%–20.70%), such products comprised 36.68%–61.02% of the edited reads generated by pDuBE2a (Supplemental Figure 9). The CBE outcomes (sole C-to-T conversions in edits) of pDuBE2a were substantially higher than those of pDuBE2b across all targets by at least 4.47-fold and significantly higher than those of pDuBE2c at four of the six targets (excluding *IPA1*-T2 and *PDS*-T1, Figure 1C, *P* < 0.05). By contrast, overall ABE activities were comparable among the constructs, with mean A-to-G conversion rates of 18.72% for pDuBE2a, 23.88% for pDuBE2b, and 11.69% for pDuBE2c (Figure 1C). These findings indicate that the architecture of pDuBE2a was most effective for the integration of Sdd7 with adenine deaminase, enabling superior dual-base-editing capabilities. The editing performance of pDuBE2a was also evaluated relative to those of three previously reported DuBEs, STEME-3, pDuBE1, and TadDE (Li et al., 2020; Xu et al., 2021; Fan et al., 2024; Yu et al., 2024), at five genomic loci using an identical vector backbone (Supplemental Figure 10). The efficiencies of simultaneous A-to-G and C-to-T conversions were significantly higher for pDuBE2a compared with STEME-3 by at least 6.42-fold across all targets and compared with pDuBE1 at four of the five targets (excluding *Pikh*-T1; Figure 1D, *P* < 0.05). For TadDE, although similar dual-editing efficiencies were observed at *BADH2*-T, *Pikh*-T1, and *SLR1*-T2, pDuBE2a still exhibited 1.99- and 18.62-fold greater A&CBE activity at *PDS*-T1 and *IPA1*-T2, respectively (Figure 1D). In addition, TadDE generated over 8.51-fold more insertions or deletions than pDuBE2a, indicating lower editing purity (Supplemental Figure 11). Thus, pDuBE2a outperformed established editors for plant dual-base editing.

Editing by pDuBE2a was next assessed at 10 endogenous targets in the rice genome in transgenic plants. Genotyping of independent T_0_ plants revealed that, on average, 49.58% of the lines contained both C-to-T and A-to-G conversions in single or multiple alleles, accounting for 78.03% of the mutants (Figure 1E; Supplemental Table 1). In these plants, A-to-G and C-to-T substitutions occurred in large editing windows between positions 3 and 11 and positions 1 and 13, respectively (Supplemental Figure 12). ABE and CBE products were found in 26.67% and 17.08% of T_0_ lines, respectively, and A&CBE products were found in 46.04% of the plants, with a maximum homozygous ratio of 20.83% at *Pikh*-T2 (Supplemental Table 1). The abundance of A&CBE lines was consistent with the higher efficiency of dual base edits in callus cells, confirming that A&CBE is the predominant activity of pDuBE2a in rice. Several T_1_ progenies derived from the edited T_0_ lines were examined, and the germline transmission of mutations confirmed the stable heritability of the pDuBE2a-induced edits (Supplemental Table 2). Because Sdd7 exhibits higher off-target activity than rAPOBEC1 (Huang et al., 2023), the editing specificity of pDuBE2a was assessed in T_0_ plants. Whole-genome sequencing revealed that pDuBE2a lines displayed comparable levels of insertions or deletions, whereas the number of single-nucleotide variations was 2.08- and 1.75-fold higher than those observed in SpCas9 and TadDE plants, respectively (Supplemental Figure 13A–13C). Notably, pDuBE2a increased both C-to-T and A-to-G single-nucleotide variations (Supplemental Figure 13D). Because Sdd7 has nonspecific double-stranded DNA-binding activity (Hwang et al., 2025), it is plausible that the resulting unintended R-loops were also deaminated by the fused TadA-8e, thereby increasing off-target A-to-G mutations in pDuBE2a-edited lines. Recent studies have shown that rational protein engineering of Sdd7, including the introduction of V132L, R119A, and R153A mutations, can reduce off-target editing without substantially compromising on-target activity. A similar strategy could be applied to pDuBE2a to enhance its specificity while maintaining high editing efficiency in future optimization efforts (Hwang et al., 2025).

To reveal the utility of pDuBE2a for artificial evolution of plant genes, an A&CBE library was used for high-throughput genetic screening of herbicide-resistant variants of the rice *OsEPSPS* gene. Using a customized BES-Designer tool (Zhou et al., 2024), a simplified array containing 160 sgRNAs was designed for *in situ* saturation mutagenesis of the 1536-bp coding sequence of *OsEPSPS* (Figure 1F). The pDuBE2a library was pooled in approximately 5000 calli and screened in culture medium supplemented with 6 mM glyphosate for 4–6 weeks (Figure 1G). A total of 43 independent resistant callus events were obtained. After the protospacer region was identified in each event, the corresponding sgRNA-associated target regions were genotyped by site-specific sequencing. As shown in Figure 1H, single amino acid mutations of Y248H, M279T, T466I, and M472T were identified in 25 events with seven types of nucleotide substitutions. Simultaneous substitutions of the Y248H-L249S, E278K-M279T, T466I-Y467C, and R471K-M472T double mutations were detected as individual edits in eight calli and occurred as independent alleles of their single-mutation counterparts in 10 additional callus events. To examine the herbicide tolerance of the library-screened variants, the eight mutations were precisely introduced into rice cells using an ePE2 prime-editing system (Li et al., 2023). To characterize the role of each point mutation, E278K, L249S, Y467C, and R471K were separately re-edited. All of the mutations were obtained in transformed cells, except for E278K, M279T, and E278K-M279T, which may be difficult to achieve because of the lack of appropriate nearby NGG PAMs for ePE2 editing. The calli of each edited event were transferred to glyphosate medium for preliminary tolerance evaluation (Supplemental Figure 14). The single mutants Y248H, T466I, and M472T and the double mutants Y248H-L249S, T466I-Y467C, and R471K-M472T were tolerant to 6 mM glyphosate, whereas the L249S, Y467C, and R471K mutants were highly sensitive. Y248H and Y248H-L249S were more tolerant than T466I, T466I-Y467C, M472T, and R471K-M472T to 12 mM glyphosate, suggesting that Y248H and Y248H-L249S may have better herbicide resistance. Interestingly, the tolerances of the Y248H-L249S, T466I-Y467C, and R471K-M472T double mutants were similar to those of the Y248H, T466I, and M472T single mutants, respectively. These results imply that Y248H, T466I, and M472T may be the key mutations associated with herbicide tolerance, whereas L249S, Y467C, and R471K are likely to be collateral neutral mutations resulting from the bystander editing activity of pDuBE2a. Structural simulations revealed that the main-effect Y248H, T466I, and M472T mutations affected the binding domain of the OsEPSPS protein and thus presumably reduced its affinity for the herbicide (Supplemental Figure 15). Because none of these mutations have been documented in rice or weeds, they appear to be novel *OsEPSPS* variants that confer resistance to glyphosate. These previously unreported variants provide further evidence for the utility of pDuBE2a in artificial evolution, as its reliable multiple-type base-editing activity facilitates the generation of abundant genetic variants.

To test the applicability of the main-effect variants, the edited T_0_ lines of Y248H, T466I, and M472T were regenerated and self-pollinated to produce offspring. We found that the homozygous T466I mutant was absent in the T_1_ generation (Supplemental Table 3), similar to the D213N mutant screened in an independent artificial evolution experiment (Zhang et al., 2023b). By contrast, homozygous Y248H and M472T mutants were obtained from the progenies of homozygous and heterozygous edited T_0_ lines (Supplemental Table 3). In this case, the homozygous Y248H and M472T mutants and the T466I heterozygous mutants were analyzed in the T_1_ generation. Compared with wild-type seedlings, the mutants showed resistance to 720 g a.i./ha Roundup (41.0% glyphosate isopropylamine salt, 0.5× the highest recommended dose in cotton fields, Figure 1I). Among the mutants, phytotoxicity was observed in the T466I and M472T lines after treatment with 1440 g a.i./ha Roundup (1× the recommended field dose), whereas the Y248H lines tolerated 2880 g a.i./ha Roundup (2× the recommended field dose). The consistent herbicide resistance observed in calli and plants suggested that Y248H might be useful for breeding of glyphosate-tolerant rice. Primary agronomic traits were investigated to evaluate the application potential of the *OsEPSPS* variants in the field. Most traits, including plant height, leaf length, and grain weight, were comparable between the wild type and the mutants (Supplemental Figure 16). However, the Y248H mutant showed a 16.67% reduction in tiller number. Because dense planting can compensate for minor reductions in tillering, the Y248H variant may still be suitable for practical breeding. By contrast, the T466I lines exhibited significantly lower seed-setting rates than other plants, confirming a fitness cost associated with reduced fertility.

Variants with multiple substitutions at different residues, such as T173I-P177S or T173I-A174V-P177S in OsEPSPS, might offer more promising herbicide tolerance (Li et al., 2016; Achary et al., 2020; Jiang et al., 2022). To increase the frequency of multiple mutations in the screen, a double sgRNA cassette was used to simultaneously edit two targets in *OsEPSPS*. Randomly paired sgRNAs were integrated to pool a library of 160 × 160 arrays (Supplemental Figure 17). Twenty-six resistance events were selected from the transformation of approximately 5000 calli, and their mutations were identified by amplicon sequencing of the complete *OsEPSPS* region. In addition to the eight editing types described above, 14 novel edits were identified, all of which had multiplex editing in the same allele of the Y248H, M279T, T466I, or M472T mutation (Supplemental Table 4). The multiplex edits were further disassembled into single amino acid substitutions. Compared with those identified in the single sgRNA library, additional glyphosate tolerance-related mutations of 15 residues were found in the double sgRNA screen (Supplemental Table 4). However, in a tentative PE-mediated assay, none of these point mutations conferred resistance in calli (Supplemental Figure 18). Although these substitutions may not directly contribute to resistance, they still provide some inspiration for the evolution of *OsEPSPS*. Together with future protein engineering, these germplasms could facilitate the development of highly resistant *OsEPSPS variants with* low fitness costs. Collectively, our results demonstrate that optimized pDuBE2a provides a powerful tool for *in situ* saturation mutagenesis and artificial evolution of agronomically important genes in rice, enabling the development of high-value novel germplasms to accelerate practical breeding.

## Funding

This work was funded by the 10.13039/501100012166National Key Research and Development Program (2022YFF1002803); the 10.13039/501100001809National Natural Science Foundation of China (32570484 and 32572441); the Science and Technology Major Project of Anhui Province (Nos. 202423m10050002, 2023n06020020, and 202423110050063); the Natural Science Foundation of Hefei (HZR2449); and the Natural Science Research Project of the Anhui Educational Committee (Nos. 2024AH050459, 2024AH050424, and 2022AH010056).

## Acknowledgments

We thank Prof. Caixia Gao at the Institute of Genetics and Developmental Biology, Chinese Academy of Sciences, for providing the sequences and plasmids of Sdd deaminases. No conflict of interest declared.

## Author contributions

J.L. and P.W. conceptualized, supervised, and supported the experiments. R.X., J.Z., and R.Q. developed the methodology. R.X., J.Z., Hui Wang, Huanhuan Wang, and X.L. performed the investigations. J.L. and R.X. wrote the original draft. P.W. and J.L. reviewed and edited the manuscript. L.P., L.Z., and J.L. provided resources.

## Supplemental information

Document S1. Supplemental Figures 1–18, Supplemental Tables 1–7, supplemental methods, and supplemental sequences

Document S2. Article plus supplemental information

## References

[bib1] Achary V.M.M., Sheri V., Manna M., Panditi V., Borphukan B., Ram B., Agarwal A., Fartyal D., Teotia D., Masakapalli S.K. (2020). Overexpression of improved EPSPS gene results in field level glyphosate tolerance and higher grain yield in rice. Plant Biotechnol. J..

[bib2] Fan T., Cheng Y., Wu Y., Liu S., Tang X., He Y., Liao S., Zheng X., Zhang T., Qi Y., Zhang Y. (2024). High performance TadA-8e derived cytosine and dual base editors with undetectable off-target effects in plants. Nat. Commun..

[bib3] Huang J., Lin Q., Fei H., He Z., Xu H., Li Y., Qu K., Han P., Gao Q., Li B. (2023). Discovery of deaminase functions by structure-based protein clustering. Cell.

[bib4] Hwang H.-Y., Lee M., Yi H., Seok C., Lim K., Na Y.R., Kang J.-S., Park J.-H., Kim D. (2025). Engineered Sdd7 cytosine base editors with enhanced specificity. Nat. Commun..

[bib5] Jiang Y., Chai Y., Qiao D., Wang J., Xin C., Sun W., Cao Z., Zhang Y., Zhou Y., Wang X.-C., Chen Q.J. (2022). Optimized prime editing efficiently generates glyphosate-resistant rice plants carrying homozygous TAP-IVS mutation in EPSPS. Mol. Plant.

[bib6] Li B., Sun C., Li J., Gao C. (2024). Targeted genome-modification tools and their advanced applications in crop breeding. Nat. Rev. Genet..

[bib7] Li C., Zhang R., Meng X., Chen S., Zong Y., Lu C., Qiu J.-L., Chen Y.-H., Li J., Gao C. (2020). Targeted, random mutagenesis of plant genes with dual cytosine and adenine base editors. Nat. Biotechnol..

[bib8] Li J., Meng X., Zong Y., Chen K., Zhang H., Liu J., Li J., Gao C. (2016). Gene replacements and insertions in rice by intron targeting using CRISPR–Cas9. Nat. Plants.

[bib9] Li J., Ding J., Zhu J., Xu R., Gu D., Liu X., Liang J., Qiu C., Wang H., Li M. (2023). Prime editing-mediated precise knockin of protein tag sequences in the rice genome. Plant Commun..

[bib10] Xu R., Kong F., Qin R., Li J., Liu X., Wei P. (2021). Development of an efficient plant dual cytosine and adenine editor. J. Integr. Plant Biol..

[bib11] Yu M., Kuang Y., Wang C., Wu X., Li S., Zhang D., Sun W., Zhou X., Ren B., Zhou H. (2024). Diverse nucleotide substitutions in rice base editing mediated by novel TadA variants. Plant Commun..

[bib12] Zhang A., Shan T., Sun Y., Chen Z., Hu J., Hu Z., Ming Z., Zhu Z., Li X., He J. (2023). Directed evolution rice genes with randomly multiplexed sgRNAs assembly of base editors. Plant Biotechnol. J..

[bib13] Zhang C., Zhong X., Li S., Yan L., Li J., He Y., Lin Y., Zhang Y., Xia L. (2023). Artificial evolution of OsEPSPS through an improved dual cytosine and adenine base editor generated a novel allele conferring rice glyphosate tolerance. J. Integr. Plant Biol..

[bib14] Zheng Z., Liu T., Chai N., Zeng D., Zhang R., Wu Y., Hang J., Liu Y., Deng Q., Tan J. (2024). PhieDBEs: a DBD-containing, PAM-flexible, high-efficiency dual base editor toolbox with wide targeting scope for use in plants. Plant Biotechnol. J..

[bib15] Zhou Q., Gao Q., Gao Y., Zhang Y., Chen Y., Li M., Wei P., Yue Z. (2025). BES-Designer: A Web Tool to Design Guide RNAs for Base Editing to Simplify Library. Interdiscip. Sci..

