# Supplementary material for: Development of dual base editors based on ssDNA-targeting SCP1.201 deaminases for the artificial evolution of novel herbicide-tolerant OsEPSPS variants
Source: Plant Commun. 2025 Dec 3;7(3):101632. doi: 10.1016/j.xplc.2025.101632 (PMC12983258; doi:10.1016/j.xplc.2025.101632)
Supplement: Document S2. Article plus supplemental information [file mmc2.pdf]

Plant Communications
Correspondence

CellPress
Partner Journal

Development of dual base editors based on ssDNA-targeting SCP1.201 deaminases for the artificial evolution of novel herbicide-tolerant *OsEPSPS* variants

Dear Editor,

Dual base editors (DuBEs) enable the simultaneous introduction of multiple types of nucleotide substitutions using a single guide RNA (sgRNA), thus greatly broadening the product diversity of base editing. Plant dual cytosine and adenine base editors (A&CBEs) were developed by fusing an apolipoprotein B mRNA-editing enzyme catalytic polypeptide (APOBEC)/activation-induced cytidine deaminase (AID)-like deaminase and an artificially evolved TadA-8e adenine deaminase to the same or opposite termini of a catalytically impaired Cas9 nickase (Li et al., 2020, 2024; Xu et al., 2021; Zhang et al., 2023a, 2023b; Zheng et al., 2024). A bifunctional TadDE derived from TadA-8e has also been used for dual C-to-T and A-to-G editing in rice (Fan et al., 2024; Yu et al., 2024). Taking advantage of these DuBEs, *in situ* targeted saturation mutagenesis and directed artificial evolution have been performed in crops to improve breeding traits (Li et al., 2024). Recently, numerous novel cytidine deaminases were identified through an AI-assisted 3D protein structure–based clustering strategy (Huang et al., 2023). Among them, a number of single-stranded DNA (ssDNA)-targeting proteins from the SCP1.201 clade (Sdds) have been engineered as compact CBEs, enabling efficient C-to-T editing at conventionally uneditable targets in crop genomes (Huang et al., 2023). However, the dual-base-editing compatibility of Sdds has not yet been assessed.

To investigate appropriate architectures for Sdd-based DuBEs, Sdd6 and Sdd7, the two most well-used and highly promising candidates (Huang et al., 2023), were separately fused to the C or N terminus of the SpCas9 (D10A) nickase (Supplemental Figure 1). The efficiency of the resulting Sdd-CBEminis was assessed at four targets (*DL*-T, *IPA1*-T1, *LAZY1*-T, and *SLR1*-T1) by amplicon next-generation sequencing of stably transformed rice calli (supplemental methods). The average cytosine conversion efficiencies of Sdd7c-CBEmini (Sdd7 fused to the C terminus of nSpCas9) and Sdd7n-CBEmini (Sdd7 fused to the N terminus) were 42.70% and 49.89%, respectively, and they outperformed their Sdd6 counterparts by 7.94- and 2.64-fold (Figure 1A). The Sdd7-CBEminis also produced more C-to-T and C-to-G edits than the Sdd6-derived editors (Supplemental Figure 2). Intriguingly, it appeared that the efficiency of the Sdd6-derived editors depended on the specific terminus to which Sdd6 was fused. The efficiency of Sdd6n-CBEmini was 2.44- to 7.08-fold higher than that of Sdd6c-CBEmini at all four targets (Supplemental Figure 3, $P < 0.05$), suggesting that Sdd6 was more effective as an N-terminal fusion. By contrast, Sdd7n-CBEmini and Sdd7c-CBEmini exhibited similar efficiencies at the *DL*-T, *SLR1*-T1, and *LAZY1*-T1 sites ($P > 0.05$), although the efficiency of Sdd7n-CBEmini was slightly higher (1.39-fold) than that of Sdd7c-CBEmini at the *IPA1*-T1 site (Supplemental Figure 3). In addition, Sdd7c- and Sdd7n-CBEmini exhibited the same editing range between positions 3 and 12 (counting from the distal end of the protospacer; Supplemental Figure 4). These results suggest that Sdd7 does not have an intrinsic terminus preference for base editing. 3D structure predictions revealed that, unlike those of Sdd6n-CBEmini, Sdd7c-CBEmini, and Sdd7n-CBEmini, the N-terminal region and α-helical domain of Sdd6 in Sdd6c-CBEmini were largely disordered and exhibited significant spatial separation from Cas9, inducing potential steric hindrance that may impair editing activity (Supplemental Figure 5).

Because the Sdd7-CBEminis exhibited better cytosine editing efficiency in the rice genome, Sdd7 was chosen for integration with TadA-8e at the same or opposite terminus of nSpCas9. The TadA-8e–Sdd7–nSpCas9–3×UGI and TadA-8e–nSpCas9–Sdd7–3×UGI fusions were designated plant DuBE2a (pDuBE2a) and pDuBE2b, respectively (Figure 1B; Supplemental Figure 6). A multiplex orthogonal base-editor architecture consisting of the nSpCas9 (D10A), MCP–Sdd7–3×UGI, TadA-8e–N22p, and sgRNA–boxB–MS2 modules was also established and designated pDuBE2c. To characterize the editing profiles of the pDuBE2s, six sgRNAs targeting multiple As and Cs were designed. Amplicon next-generation sequencing revealed that pDuBE2a, pDuBE2b, and pDuBE2c produced targeted mutations in 58.02%, 27.08%, and 15.33% of reads, respectively, most of which were C-to-T and/or A-to-G conversions (Supplemental Figure 7). The overall base-editing efficiency of pDuBE2a was significantly higher than that of pDuBE2b at five of the six sites (excluding *Pikh*-T1) by 1.81- to 7.06-fold and higher than that of pDuBE2c at all targets by 1.67- to 41.86-fold ($P < 0.05$, Figure 1C). In terms of pDuBE2a outcomes, edits carrying C-to-T conversions accounted for 27.43% to 43.06% of the reads, much higher than the 0%–7.67% of pDuBE2b and the 0%–6.78% of pDuBE2c ($P < 0.05$, Supplemental Figure 8). The average efficiency of A-to-G conversions produced by pDuBE2a was 46.78%, 1.84- and 3.62-fold higher than the efficiencies of pDuBE2b and pDuBE2c, respectively ($P < 0.05$). The dual A-to-G and C-to-T editing efficiency of pDuBE2a ranged from 20.58% to 39.39% and was significantly higher than that of pDuBE2b and pDuBE2c by at least 5.62-fold (Figure 1C, $P < 0.05$). In contrast to the low proportions of A&CBE products generated by pDuBE2b (0%–11.83%) and pDuBE2c (0%–20.70%), such products comprised 36.68%–61.

 © 2025 The Author(s). Published by Elsevier Inc. on behalf of CAS Center for Excellence in Molecular Plant Sciences, Chinese Academy of Sciences, Chinese Society for Plant Biology.
This is an open access article under the CC BY license (http://creativecommons.org/licenses/by/4.0/).

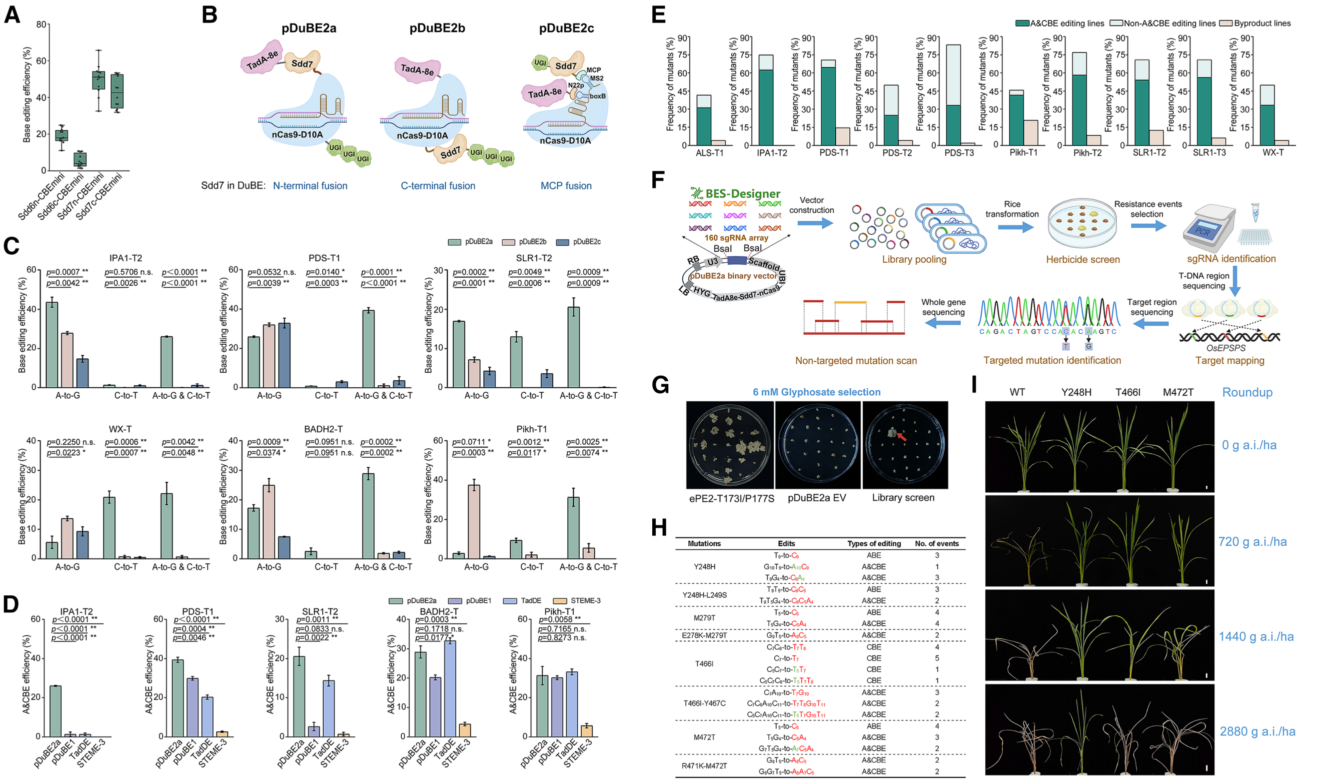

Figure 1. Editing and applications of Sdd-derived dual base editors in rice.

(A) Base-editing efficiencies of Sdd6- and Sdd7-derived CBEminis. Efficiency was expressed as the ratio of the cytosine-edited reads to total reads. Boxplots show the editing at four endogenous targets with three biological replicates.

(B) Diagram of Sdd7 dual base editors. pDuBE2a, both Sdd7 and TadA-8e fused to the N-terminus of an SpCas9 D10A nickase (Cas9-D10A), followed by C-terminal UGIs; pDuBE2b, Sdd7 and TadA-8e fused to the opposite terminus of Cas9-D10A; pDuBE2c, MS2, and boxB integrated into the sgRNA scaffold to recruit MCP-Sdd7-UGI and N22p-TadA-8e, respectively.

(C) Base-editing efficiency of pDuBE2s in rice calli. A-to-G, C-to-T, and concurrent A-to-G and C-to-T conversions are defined as ABE, CBE, and A&CBE outcomes, respectively, of the dual base editors. Mean efficiencies and standard deviations are presented (n = 3). P values were determined by one-way ANOVA. $^{**}P < 0.01$, $^{*}P < 0.05$, and n.s., not significant.

(D) Editing efficiencies of the plant dual base editors pDuBE2a, pDuBE1, TadDE, and STEME-3 were quantified by amplicon sequencing. The A&CBE editing efficiency was calculated as the percentage of sequencing reads with concurrent A-to-G and C-to-T substitutions relative to the total number of clean reads. Values shown are the mean ± standard deviation from three biological replicates across five independent genomic target sites.

(E) Base editing of pDuBE2a in T_0 transgenic rice plants. The ratios of edited lines are presented. To ensure heritability, a threshold of 15% in the Hi-TOM (high-throughput tracking of mutations) assay was used to filter the edits. Lines with A&CBE edits (simultaneous A-to-G and C-to-T editing in one allele) are shown in dark turquoise, and lines with only individual A-to-G and/or C-to-T edits are shown in light turquoise. Plants with byproducts are shown in light peach.

(F) Schematic illustration of the procedure for evolving glyphosate-resistant *OsEPSPS* mutants with the pDuBE2a library. A simplified array of 160 sgRNAs was designed for saturation mutagenesis of *OsEPSPS* with the BES-Designer tool. The array was assembled into pDuBE2a to pool a dual-editing library for rice transformation. The T-DNA regions of glyphosate-tolerant callus events were genotyped, and the mutations were identified. The complete *OsEPSPS* gene was then scanned to identify any unintended mutations in the remaining sequence by potential bystander editing or transient expression of unintegrated sgRNA.

(G) Representative screen of glyphosate-resistant calli. Transformation with the ePE2 (prime editing) vector to induce a T173I-P177S mutation in *OsEPSPS* was used as a positive control for glyphosate selection, and transformation with the pDuBE2a empty vector (EV) was used as a negative control. Calli pooled with the pDuBE2a library were screened by 6 mM glyphosate selection for 4–6 weeks, and a representative plate is shown. The red arrow highlights a resistant callus.

(H) Glyphosate-resistant *OsEPSPS* mutants screened by pDuBE2a with the 160-sgRNA array. From left to right, mutated residues identified in resistant calli; base edits of *OsEPSPS* in resistant calli: missense mutations resulting in amino acid changes are highlighted in red, synonymous substitutions of adjacent residues are shown in green, and the numbers indicate the positions of the edited nucleotides within the target sequences; types of pDuBE2a activity that produced the edits; and numbers of callus events harboring the mutations. Some events may be chimeras of different variants.

(I) Herbicide resistance of library-screened mutations in the T_1 generation. The mutants were reproduced by prime editing to ensure precise mutations. Three-week-old T_1 heterozygous lines of T466I and T_1 homozygous lines of Y248H or M472T (as described in Supplemental Table 2) were sprayed with serial dilutions of the commercial herbicide Roundup. The plants were imaged after 2 weeks of incubation. Scale bar, 1 cm.

02% of the edited reads generated by pDuBE2a (Supplemental Figure 9). The CBE outcomes (sole C-to-T conversions in edits) of pDuBE2a were substantially higher than those of pDuBE2b across all targets by at least 4.47-fold and significantly higher than those of pDuBE2c at four of the six targets (excluding *IPA1*-T2 and *PDS*-T1, Figure 1C, $P < 0.05$). By contrast, overall ABE activities were comparable among the constructs, with mean A-to-G conversion rates of 18.72% for pDuBE2a, 23.88% for pDuBE2b, and 11.69% for pDuBE2c (Figure 1C). These findings indicate that the architecture of pDuBE2a was most effective for the integration of Sdd7 with adenine deaminase, enabling superior dual-base-editing capabilities. The editing performance of pDuBE2a was also evaluated relative to those of three previously reported DuBEs, STEME-3, pDuBE1, and TadDE (Li et al., 2020; Xu et al., 2021; Fan et al., 2024; Yu et al., 2024), at five genomic loci using an identical vector backbone (Supplemental Figure 10). The efficiencies of simultaneous A-to-G and C-to-T conversions were significantly higher for pDuBE2a compared with STEME-3 by at least 6.42-fold across all targets and compared with pDuBE1 at four of the five targets (excluding *Pikh*-T1; Figure 1D, $P < 0.05$). For TadDE, although similar dual-editing efficiencies were observed at *BADH2*-T, *Pikh*-T1, and *SLR1*-T2, pDuBE2a still exhibited 1.99- and 18.62-fold greater A&CBE activity at *PDS*-T1 and *IPA1*-T2, respectively (Figure 1D). In addition, TadDE generated over 8.51-fold more insertions or deletions than pDuBE2a, indicating lower editing purity (Supplemental Figure 11). Thus, pDuBE2a outperformed established editors for plant dual-base editing.

Editing by pDuBE2a was next assessed at 10 endogenous targets in the rice genome in transgenic plants. Genotyping of independent T_0 plants revealed that, on average, 49.58% of the lines contained both C-to-T and A-to-G conversions in single or multiple alleles, accounting for 78.03% of the mutants (Figure 1E; Supplemental Table 1). In these plants, A-to-G and C-to-T substitutions occurred in large editing windows between positions 3 and 11 and positions 1 and 13, respectively (Supplemental Figure 12). ABE and CBE products were found in 26.67% and 17.08% of T_0 lines, respectively, and A&CBE products were found in 46.04% of the plants, with a maximum homozygous ratio of 20.83% at *Pikh*-T2 (Supplemental Table 1). The abundance of A&CBE lines was consistent with the higher efficiency of dual base edits in callus cells, confirming that A&CBE is the predominant activity of pDuBE2a in rice. Several T_1 progenies derived from the edited T_0 lines were examined, and the germline transmission of mutations confirmed the stable heritability of the pDuBE2a-induced edits (Supplemental Table 2). Because Sdd7 exhibits higher off-target activity than rAPOBEC1 (Huang et al., 2023), the editing specificity of pDuBE2a was assessed in T_0 plants. Whole-genome sequencing revealed that pDuBE2a lines displayed comparable levels of insertions or deletions, whereas the number of single-nucleotide variations was 2.08- and 1.75-fold higher than those observed in SpCas9 and TadDE plants, respectively (Supplemental Figure 13A–13C). Notably, pDuBE2a increased both C-to-T and A-to-G single-nucleotide variations (Supplemental Figure 13D). Because Sdd7 has nonspecific double-stranded DNA-binding activity (Hwang et al., 2025), it is plausible that the resulting unintended R-loops were also deaminated by the fused TadA-8e, thereby increasing off-target A-to-G mutations in pDuBE2a-edited lines. Recent studies have shown that rational protein engineering of Sdd7, including the introduction of V132L, R119A, and R153A mutations, can reduce off-target editing without substantially compromising on-target activity. A similar strategy could be applied to pDuBE2a to enhance its specificity while maintaining high editing efficiency in future optimization efforts (Hwang et al., 2025).

To reveal the utility of pDuBE2a for artificial evolution of plant genes, an A&CBE library was used for high-throughput genetic screening of herbicide-resistant variants of the rice *OsEPSPS* gene. Using a customized BES-Designer tool (Zhou et al., 2024), a simplified array containing 160 sgRNAs was designed for *in situ* saturation mutagenesis of the 1536-bp coding sequence of *OsEPSPS* (Figure 1F). The pDuBE2a library was pooled in approximately 5000 calli and screened in culture medium supplemented with 6 mM glyphosate for 4–6 weeks (Figure 1G). A total of 43 independent resistant callus events were obtained. After the protospacer region was identified in each event, the corresponding sgRNA-associated target regions were genotyped by site-specific sequencing. As shown in Figure 1H, single amino acid mutations of Y248H, M279T, T466I, and M472T were identified in 25 events with seven types of nucleotide substitutions. Simultaneous substitutions of the Y248H-L249S, E278K-M279T, T466I-Y467C, and R471K-M472T double mutations were detected as individual edits in eight calli and occurred as independent alleles of their single-mutation counterparts in 10 additional callus events. To examine the herbicide tolerance of the library-screened variants, the eight mutations were precisely introduced into rice cells using an ePE2 prime-editing system (Li et al., 2023). To characterize the role of each point mutation, E278K, L249S, Y467C, and R471K were separately re-edited. All of the mutations were obtained in transformed cells, except for E278K, M279T, and E278K-M279T, which may be difficult to achieve because of the lack of appropriate nearby NGG PAMs for ePE2 editing. The calli of each edited event were transferred to glyphosate medium for preliminary tolerance evaluation (Supplemental Figure 14). The single mutants Y248H, T466I, and M472T and the double mutants Y248H-L249S, T466I-Y467C, and R471K-M472T were tolerant to 6 mM glyphosate, whereas the L249S, Y467C, and R471K mutants were highly sensitive. Y248H and Y248H-L249S were more tolerant than T466I, T466I-Y467C, M472T, and R471K-M472T to 12 mM glyphosate, suggesting that Y248H and Y248H-L249S may have better herbicide resistance. Interestingly, the tolerances of the Y248H-L249S, T466I-Y467C, and R471K-M472T double mutants were similar to those of the Y248H, T466I, and M472T single mutants, respectively. These results imply that Y248H, T466I, and M472T may be the key mutations associated with herbicide tolerance, whereas L249S, Y467C, and R471K are likely to be collateral neutral mutations resulting from the bystander editing activity of pDuBE2a. Structural simulations revealed that the main-effect Y248H, T466I, and M472T mutations affected the binding domain of the OsEPSPS protein and thus presumably reduced its affinity for the herbicide (Supplemental Figure 15). Because none of these mutations have been documented in rice or weeds, they appear to be novel *OsEPSPS* variants that confer resistance to glyphosate. These previously unreported variants provide further evidence for the utility of pDuBE2a in artificial evolution, as

its reliable multiple-type base-editing activity facilitates the generation of abundant genetic variants.

To test the applicability of the main-effect variants, the edited T_0 lines of Y248H, T466I, and M472T were regenerated and self-pollinated to produce offspring. We found that the homozygous T466I mutant was absent in the T_1 generation (Supplemental Table 3), similar to the D213N mutant screened in an independent artificial evolution experiment (Zhang et al., 2023b). By contrast, homozygous Y248H and M472T mutants were obtained from the progenies of homozygous and heterozygous edited T_0 lines (Supplemental Table 3). In this case, the homozygous Y248H and M472T mutants and the T466I heterozygous mutants were analyzed in the T_1 generation. Compared with wild-type seedlings, the mutants showed resistance to 720 g a.i./ha Roundup (41.0% glyphosate isopropylamine salt, 0.5× the highest recommended dose in cotton fields, Figure 1I). Among the mutants, phytotoxicity was observed in the T466I and M472T lines after treatment with 1440 g a.i./ha Roundup (1× the recommended field dose), whereas the Y248H lines tolerated 2880 g a.i./ha Roundup (2× the recommended field dose). The consistent herbicide resistance observed in calli and plants suggested that Y248H might be useful for breeding of glyphosate-tolerant rice. Primary agronomic traits were investigated to evaluate the application potential of the *OsEPSPS* variants in the field. Most traits, including plant height, leaf length, and grain weight, were comparable between the wild type and the mutants (Supplemental Figure 16). However, the Y248H mutant showed a 16.67% reduction in tiller number. Because dense planting can compensate for minor reductions in tillering, the Y248H variant may still be suitable for practical breeding. By contrast, the T466I lines exhibited significantly lower seed-setting rates than other plants, confirming a fitness cost associated with reduced fertility.

Variants with multiple substitutions at different residues, such as T173I-P177S or T173I-A174V-P177S in OsEPSPS, might offer more promising herbicide tolerance (Li et al., 2016; Achary et al., 2020; Jiang et al., 2022). To increase the frequency of multiple mutations in the screen, a double sgRNA cassette was used to simultaneously edit two targets in *OsEPSPS*. Randomly paired sgRNAs were integrated to pool a library of 160 × 160 arrays (Supplemental Figure 17). Twenty-six resistance events were selected from the transformation of approximately 5000 calli, and their mutations were identified by amplicon sequencing of the complete *OsEPSPS* region. In addition to the eight editing types described above, 14 novel edits were identified, all of which had multiplex editing in the same allele of the Y248H, M279T, T466I, or M472T mutation (Supplemental Table 4). The multiplex edits were further disassembled into single amino acid substitutions. Compared with those identified in the single sgRNA library, additional glyphosate tolerance-related mutations of 15 residues were found in the double sgRNA screen (Supplemental Table 4). However, in a tentative PE-mediated assay, none of these point mutations conferred resistance in calli (Supplemental Figure 18). Although these substitutions may not directly contribute to resistance, they still provide some inspiration for the evolution of *OsEPSPS*. Together with future protein engineering, these germplasms could facilitate the development of highly resistant *OsEPSPS variants with* low fitness costs. Collectively, our results demonstrate that optimized pDuBE2a provides a powerful tool for *in situ* saturation mutagenesis and artificial evolution of agronomically important genes in rice, enabling the development of high-value novel germplasms to accelerate practical breeding.

FUNDING

This work was funded by the National Key Research and Development Program (2022YFF1002803); the National Natural Science Foundation of China (32570484 and 32572441); the Science and Technology Major Project of Anhui Province (Nos. 202423m10050002, 2023n06020020, and 202423110050063); the Natural Science Foundation of Hefei (HZR2449); and the Natural Science Research Project of the Anhui Educational Committee (Nos. 2024AH050459, 2024AH050424, and 2022AH010056).

ACKNOWLEDGMENTS

We thank Prof. Caixia Gao at the Institute of Genetics and Developmental Biology, Chinese Academy of Sciences, for providing the sequences and plasmids of Sdd deaminases. No conflict of interest declared.

AUTHOR CONTRIBUTIONS

J.L. and P.W. conceptualized, supervised, and supported the experiments. R.X., J.Z., and R.Q. developed the methodology. R.X., J.Z., Hui Wang, Huanhuan Wang, and X.L. performed the investigations. J.L. and R.X. wrote the original draft. P.W. and J.L. reviewed and edited the manuscript. L.P., L.Z., and J.L. provided resources.

SUPPLEMENTAL INFORMATION

Supplemental information is available at *Plant Communications Online*.

Received: April 5, 2025
Revised: October 25, 2025
Accepted: December 1, 2025
Published: December 3, 2025

Rongfang Xu[1,5], Jiahui Zhu[1,2,3,5], Hui Wang[2,3], Huanhuan Wang[1,2], Liangxia Zhao[2], Xiaoshuang Liu[2], Ruiying Qin[1], Lang Pan[4], Pengcheng Wei[2,3,] and Juan Li[1,*]*

[1]Anhui Province Key Laboratory of Rice Germplasm Innovation and Molecular Improvement, Anhui Academy of Agricultural Sciences, Hefei 230031, P.R. China
[2]College of Agronomy, Anhui Agricultural University, Hefei 230036, P.R. China
[3]Research Centre for Biological Breeding Technology, Advanced Academy, Anhui Agricultural University, Hefei 230036, P.R. China
[4]College of Plant Protection, Hunan Agricultural University, Changsha 410128, P.R. China
[5]These authors contributed equally to this article.
***Correspondence: Pengcheng Wei (weipengcheng@gmail.com), Juan Li (lijuan@aaas.org.cn)**
https://doi.org/10.1016/j.xplc.2025.101632

REFERENCES

Achary, V.M.M., Sheri, V., Manna, M., Panditi, V., Borphukan, B., Ram, B., Agarwal, A., Fartyal, D., Teotia, D., Masakapalli, S.K., et al. (2020). Overexpression of improved EPSPS gene results in field level glyphosate tolerance and higher grain yield in rice. Plant Biotechnol. J. **18**:2504–2519. https://doi.org/10.1111/pbi.13428.

Fan, T., Cheng, Y., Wu, Y., Liu, S., Tang, X., He, Y., Liao, S., Zheng, X., Zhang, T., Qi, Y., and Zhang, Y. (2024). High performance TadA-8e derived cytosine and dual base editors with undetectable off-target effects in plants. Nat. Commun. **15**:5103. https://doi.org/10.1038/s41467-024-49473-w.

Huang, J., Lin, Q., Fei, H., He, Z., Xu, H., Li, Y., Qu, K., Han, P., Gao, Q., Li, B., et al. (2023). Discovery of deaminase functions by structure-based protein clustering. Cell **186**:3182–3195.e14.

Hwang, H.-Y., Lee, M., Yi, H., Seok, C., Lim, K., Na, Y.R., Kang, J.-S., Park, J.-H., and Kim, D. (2025). Engineered Sdd7 cytosine base editors with enhanced specificity. Nat. Commun. **16**:5881. https://doi.org/10.1038/s41467-025-60789-z.

Jiang, Y., Chai, Y., Qiao, D., Wang, J., Xin, C., Sun, W., Cao, Z., Zhang, Y., Zhou, Y., Wang, X.-C., and Chen, Q.J. (2022). Optimized prime editing efficiently generates glyphosate-resistant rice plants carrying homozygous TAP-IVS mutation in EPSPS. Mol. Plant **15**:1646–1649. https://doi.org/10.1016/j.molp.2022.09.006.

Li, B., Sun, C., Li, J., and Gao, C. (2024). Targeted genome-modification tools and their advanced applications in crop breeding. Nat. Rev. Genet. **25**:603–622. https://doi.org/10.1038/s41576-024-00720-2.

Li, C., Zhang, R., Meng, X., Chen, S., Zong, Y., Lu, C., Qiu, J.-L., Chen, Y.-H., Li, J., and Gao, C. (2020). Targeted, random mutagenesis of plant genes with dual cytosine and adenine base editors. Nat. Biotechnol. **38**:875–882. https://doi.org/10.1038/s41587-019-0393-7.

Li, J., Meng, X., Zong, Y., Chen, K., Zhang, H., Liu, J., Li, J., and Gao, C. (2016). Gene replacements and insertions in rice by intron targeting using CRISPR–Cas9. Nat. Plants **2**:16139. https://doi.org/10.1038/nplants.2016.139.

Li, J., Ding, J., Zhu, J., Xu, R., Gu, D., Liu, X., Liang, J., Qiu, C., Wang, H., Li, M., et al. (2023). Prime editing-mediated precise knockin of protein tag sequences in the rice genome. Plant Commun. **4**:100572.

Xu, R., Kong, F., Qin, R., Li, J., Liu, X., and Wei, P. (2021). Development of an efficient plant dual cytosine and adenine editor. J. Integr. Plant Biol. **63**:1600–1605. https://doi.org/10.1111/jipb.13146.

Yu, M., Kuang, Y., Wang, C., Wu, X., Li, S., Zhang, D., Sun, W., Zhou, X., Ren, B., and Zhou, H. (2024). Diverse nucleotide substitutions in rice base editing mediated by novel TadA variants. Plant Commun. **5**:100926. https://doi.org/10.1016/j.xplc.2024.100926.

Zhang, A., Shan, T., Sun, Y., Chen, Z., Hu, J., Hu, Z., Ming, Z., Zhu, Z., Li, X., He, J., et al. (2023a). Directed evolution rice genes with randomly multiplexed sgRNAs assembly of base editors. Plant Biotechnol. J. **21**:2597–2610. https://doi.org/10.1111/pbi.14156.

Zhang, C., Zhong, X., Li, S., Yan, L., Li, J., He, Y., Lin, Y., Zhang, Y., and Xia, L. (2023b). Artificial evolution of OsEPSPS through an improved dual cytosine and adenine base editor generated a novel allele conferring rice glyphosate tolerance. J. Integr. Plant Biol. **65**:2194–2203. https://doi.org/10.1111/jipb.13543.

Zheng, Z., Liu, T., Chai, N., Zeng, D., Zhang, R., Wu, Y., Hang, J., Liu, Y., Deng, Q., and Tan, J. (2024). PhieDBEs: a DBD-containing, PAM-flexible, high-efficiency dual base editor toolbox with wide targeting scope for use in plants. Plant Biotechnol. J. **22**:3164–3174.

Zhou, Q., Gao, Q., Gao, Y., Zhang, Y., Chen, Y., Li, M., Wei, P., and Yue, Z. (2025). BES-Designer: A Web Tool to Design Guide RNAs for Base Editing to Simplify Library. Interdiscip. Sci. **17**:134–139. https://doi.org/10.1007/s12539-024-00663-6.

Plant Communications, Volume *7*

Supplemental information

Development of dual base editors based on ssDNA-targeting SCP1.201 deaminases for the artificial evolution of novel herbicide-tolerant *OsEPSPS* variants

Rongfang Xu, Jiahui Zhu, Hui Wang, Huanhuan Wang, Liangxia Zhao, Xiaoshuang Liu, Ruiying Qin, Lang Pan, Pengcheng Wei, and Juan Li

Development of dual base editors based on ssDNA-targeting SCP1.201 deaminases for the artificial evolution of novel herbicide-tolerance *OsEPSPS* variants

Rongfang Xu[1#], Jiahui Zhu[1,2,3#], Hui Wang[2,3], Huanhuan Wang[1,2], Liangxia Zhao[2], Xiaoshuang Liu[2], Ruiying Qin[1], Lang Pan[4], Pengcheng Wei[2,3*], Juan Li[1*]

1, Anhui Province Key Laboratory of Rice Germplasm Innovation and Molecular Improvement, Anhui Academy of Agricultural Sciences, Hefei, 230031, P. R. China;

2, College of Agronomy, Anhui Agricultural University, Hefei, 230036, P. R. China;

3, Research Centre for Biological Breeding Technology, Advance Academy, Anhui Agricultural University, Hefei, 230036, P. R. China;

4, College of Plant Protection, Hunan Agricultural University, Changsha, 410128, P. R. China.

#These authors contributed equally to this paper;

* Corresponding authors: Dr. Pengcheng Wei weipengcheng@gmail.com or Dr. Juan Li lijuan@aaas.org.cn.

Running title: Development of DuBE from Sdd for the evolution of OsEPSPS.

Keywords: Base editing; deaminase; library screen; glyphosate; *OsEPSPS* variants;

Supplemental materials.

Supplemental methods.

Supplemental Figure 1. Expression cassettes of the Sdd-derived CBEminis.

Supplemental Figure 2. Cytosine conversion efficiencies of the CBEminis.

Supplemental Figure 3. Base editing efficiency of Sdd-CBEminis in stably transformed rice cells.

Supplemental Figure 4. Heatmap of base editing efficiencies induced by Sdd7n- and Sdd7c-CBEmini in calli.

Supplemental Figure 5. Three-dimensional protein structures of the Sdd-CBEminis.

Supplemental Figure 6. Expression cassette structures of Sdd7-derived dual base editors.

Supplemental Figure 7. Efficiency of pDuBE2s editing outcomes in rice calli.

Supplemental Figure 8. Base editing profile of the pDuBE2s in rice calli.

Supplemental Figure 9. A&CBE purity of pDuBE2s outcomes in rice calli.

Supplemental Figure 10. Illustrations of the established plant dual base editors for the efficiency comparison with pDuBE2a.

Supplemental Figure 11. Efficiencies of non-A&CBE outcomes of the plant dual base editors in rice calli.

Supplemental Figure 12. ABE and CBE editing frequency of pDuBE2a in transgenic rice plants.

Supplemental Figure 13. Genome-wide off-target assessments of pDuBE2a in T_0 transgenic rice.

Supplemental Figure 14. Glyphosate tolerance of rice calli harboring OsEPSPS mutations.

Supplemental Figure 15. Structural model of the OsEPSPS and molecular interactions with glyphosate.

Supplemental Figure 16. Phenotypic analysis of screened OsEPSPS mutants.

Supplemental Figure 17. Schematic illustration of double sgRNA array construction.

Supplemental Figure 18. Glyphosate tolerance identification of the mutations disassembled from double sgRNA library-screened complex edits.

Supplemental Table 1. Base editing of pDuBE2a in T0 transgenic rice plants.

Supplemental Table 2. Transmission of edits and T-DNA in the T1 generation of pDuBE2a.

Supplemental Table 3. Inheritance of the point mutations in rice.

Supplemental Table 4. Glyphosate-resistant OsEPSPS mutations screened by the pDuBE2a library with the double-sgRNA array.

Supplemental Table 5. DuBE-editing related oligos in the study.

Supplemental Table 6. Simplified sgRNA array of the OsEPSPS coding region.

Supplemental Table 7. epegRNAs designed to induce single or double mutations using the ePE2 system.

Supplemental sequences.

Supplemental methods

Vector constructions

The sequences of Sdd6 and Sdd7 were codon optimized for rice expression and synthesized (GenScript, Nanjing, China). The nSpCas9 sequence together with the N- or C-terminal 32-aa linker was amplified from the CGBEco vector (Jiang et al., 2025), and then assembled with Sdd6/Sdd7 via a HiFi DNA Assembly Kit (NEB, Ipswich, USA). The fusions were confirmed by Sanger sequencing (Sangon Biotech, Shanghai, China). The Sdd-mini fusions were subsequently inserted downstream of the ZmUBI promoter in the pHUC400 backbone using a *Pst*I/*Sac*I double digestion (Li et al., 2021). An OsU3-driven sgRNA expression cassette from pHUC411 was inserted into the *Hind*III site for gRNA integration.

To examine the dual editing capability of Sdds, the sequences of TadA-8e and triplet copies of UGI were amplified from pHUC411-ABE8e and pHUC-eA3A, respectively (Li *et al.*, 2021). The sequences were subsequently assembled into Sdd7n-mini or Sdd7c-mini to construct pDuBE2a/2b fusions. To develop the pDuBE2c system, MCP and N22p were synthesized and assembled with nSpCas9, Sdd7, TadA-8e, and UGI following the previously reported MoBE architecture (Zhang et al., 2023). In addition, the MS2 and boxB RNA aptamers were engineered to the 3' end of the sgRNA scaffold. A chimeric sequence was integrated between the tRNA and HDV ribozyme

sequences from the ePE2 vector (Li et al., 2023), which was then used to replace the U3 cassette in the backbone by *Hind*III digestion for effective and precise sgRNA expression. For efficiency comparisons, a quintuple mutation of R26G/V28A/A48R/Y73S/H96N were introduced into TadA-8e as TadA-dual. Then, TadA-dual was fused to nSpCas9-UGIs as TadDE (Neugebauer et al., 2023). Meanwhile, the sequence of human APOBECA3a and ecTadA-TadA7.10 were cloned from SpCas9-eA3A and pHUN411-ABE (Hao et al., 2019; Li *et al.*, 2021), respectively, for assembling STEME-3. The fusion of pDuBE1, TadDE, and STEME-3 were incorporated into the same backbone of pDuBE2a through *Pst*I/*Sac*I double digestions.

To assess the editing of the Sdd-derived base editors, sgRNAs containing multiple Cs and/or As were randomly designed in the following endogenous rice genes: *OsDL* (*LOC_Os03g11600*), *OsIPA1* (*LOC_Os08g39890*), *OsLAZY1* (*LOC_Os11g29840*), *OsSLR1* (*LOC_Os03g49990*), *OsPDS* (*LOC_Os03g08570*), *OsALS1* (*LOC_Os02g30630*), *OsBADH2* (*LOC_Os08g32870*), *OsWX* (*LOC_Os06g04200*), and *OsPikh* (*LOC_Os11g42010*). The construction of sgRNAs in the Sdd-derived editing vectors followed a previous protocol (Li et al., 2021). The adapter-attached oligos of the forward and reverse strands of the protospacer were annealed and assembled into *Bsa*I-predigested binary vectors to replace the spectinomycin gene through Gibson cloning. The primers and oligos used are listed in Supplemental Table 5.

Rice transformation and editing profile assessments

The binary vectors were introduced into *Agrobacterium tumefaciens* strain EHA105. PCR-positive clones were verified via Sanger sequencing of the protospacer region (Sangon, Shanghai, China). To assess editing efficiency, three independent Agrobacterium clones of each vector were separately cultured as biological replicates for callus infection.

Rice transformation was performed following a standard protocol with minor modifications (Hu et al., 2016). Embryos were excised from mature seeds of *Oryza sativa* ssp. *japonica* cv. Nipponbare for two weeks to induce callus formation. Explants were pre-cultured for five days to generate secondary calli, which were subsequently infected with bacterial suspensions for 15 minutes. For each transformant, 300–350 calli were cultured in medium supplemented with 50 mg/L hygromycin for three weeks. Resistant calli were transferred to regeneration medium supplemented with 25 mg/L hygromycin for an additional four weeks. The plant materials were incubated in a growth chamber at 30°C with a 16-hour light/8-hour dark photoperiod. To determine agronomic traits, the plants were grown in 2025 summer at field of Hefei (31° 51′N, 117°15′″E).

The editing efficiencies of base editors in rice cells were assessed via next-generation sequencing (NGS) according to a previously established pipeline (Liu et al., 2024). The assessment was applied in two-week-selected

calli population. Approximately 200 hygromycin-resistant independent events were collected to represent cell populations. For each event, a newly emerged callus was selected. The target regions were amplified via Phanta UniFi Master Mix (Vazyme Biotech, Nanjing, China), and the amplicons were sequenced on an Illumina HiSeq X Ten platform with a paired-end 150 bp (PE-150) pattern (Azenta Life Sciences, Suzhou, China). The NGS data are available in the National Genomics Data Center under BioProject number PRJNA1244604. Editing outcomes were analyzed using the CRISPResso2 program with the “Base editor” model (Clement et al., 2019).

To genotype transgenic plants, leaves from at least three different tillers of an independent line were collected for genomic DNA extraction. Target amplicons from individual lines were analyzed using the high-throughput tracking of mutations (Hi-TOM) assay with a 15% threshold (Sun et al., 2024). The primers used in this study are listed in supplemental Table 5.

Off-target assessment

To investigate genome-wide off-target effects, a total of twenty-four edited plants of DuBE2a and TadDE were selected for whole-genome sequencing (WGS). Six plants of SpCas9 were used as control. Nine wild-type plants were used for filtering background mutations. The genomic DNA was extracted from leaves of 4~6-week-old T_0 line plants or 14-day-old seedlings via the High-Performance Plant Genomic DNA Extraction Kit (TIANGEN, Beijing, China). All samples were sequencing on the DNB SEQ platform (BGI,

Shenzheng, China), which produced an average of 22 Gb of clean reads per sample with an average depth of 51×. Variants were analyzed following a previously described protocol (Jiang *et al.*, 2025), with minor modifications that use nf-core/sarek (v3.5.1) as the detection pipeline. Briefly, the raw reads were first filtered and trimmed by fastp (v0.23.4). Clean reads were then aligned to the Nipponbare genome (https://rice.uga.edu/) with BWA-mem2 (v2.2.1). Following the GATK4 best practices, the mapping results were sorted, duplicates marked and base recalibrated via samtools (v1.21), GATK (v4.5.0.0), MarkDuplicates, and BaseRecalibrator, respectively. The mapping rates of reads ranged from 98.89% to 99.89% among all the samples, which covers the reference genome between 99.62% and 99.74%. Somatic variant calling was performed with GATK Mutect2 (v4.5.0.0), LoFreq (v2.1.5), and Strelka2 (v2.9.10), with all the programs for SNVs detection, Mutect2 and Strelka2 for InDels detection. Mutations called by all the tools were merged to obtain the high confident SNVs and InDels.

Library pooling and mutation screening

sgRNAs for the simplified array were designed using a BES-designer tool with a canonical 'NGG' PAM in the coding region of *OsEPSPS* (*LOC_Os06g04280.1*) (Zhou et al., 2024). The sequences of the protospacers are provided in Supplemental Table 6. For each 20-bp protospacer, an oligo of the forward sequence with a 5' GGCA adapter and a reverse-complement oligo with a 5' AAAC adapter were synthesized (GenScript, Nanjing, China)

and annealed to a double-stranded oligo (10 μM). Equal amounts of annealed oligos were pooled and aliquoted into 20 μL. The pooling procedure followed our previous method (Xu et al., 2021). In the pDuBE2a binary vector, a ccdB toxin gene was preplaced between *OsU3* promoter and sgRNA scaffold to avoid self-ligation. Double-stranded oligo mixtures (20 μL) were ligated with *Bsa*I-digested pDuBE2a (600 ng) using T_4 ligase at 4°C for 16 hours. Ligation products were divided into 2.5 μL aliquots and each was transformed into 100 μL DH5α chemically competent cells (transformation efficiency > 10^9 cfu/μg). After overnight growth, the clone library was extracted using a FastPure Enhanced EndoFree Plasmid Maxi Plus Kit (Vazyme Biotech, Nanjing, China). The clone library was then evaluated by amplicon NGS (PE-150, 0.5 Gb data) to confirm the minimal abundance of empty vectors (< 10%) and the presence of all sgRNAs (random member > 0.1%). Pooled plasmids were introduced into an Agrobacterium library (> 3000 clones) for rice transformation. After seven days of hygromycin selection, infected calli were transferred to medium supplemented with 6 mM glyphosate. After 4–6 weeks of selection, obviously enlarged calli were collected for genotyping. Protospacers from resistance events were identified by sequencing the T-DNA region. The sgRNA-targeting region was analyzed using site-specific primers through the Hi-TOM assay. For all resistance events, the entire genomic region of *OsEPSPS* was scanned by Sanger sequencing to validate targeted mutations and to detect any bystander mutations outside the sgRNA-targeting region.

To construct the double-sgRNA library, forward and reverse-complement sequences of protospacers were synthesized into primers for amplification of a gRNA scaffold-OsU6 fragment (supplemental sequences) with *Bsa*I restriction sites at both ends. The forward and reverse primers were mixed, and PCR was performed using Phanta Flash Super-Fidelity DNA Polymerase. The amplicons were purified using the TIANquick mini purification system and then digested with *Bsa*I for 3 hours. The products (100 ng) were inserted into predigested pDUBE2a (300 ng) to pool the libraries following the aforementioned procedure.

The genomic DNA was extracted from the resistant calli to identify the sgRNAs by sequencing the T-DNA region. Moreover, the genomic region of *OsEPSPS* was amplified and subcloned and inserted into the pEASY-Blunt T vector (TransGen Biotech, Beijing, China). Subsequentially, sequencing of PCR clones was performed to distinguish mutations at the same allele or separate alleles.

Herbicide tolerance identification of screened mutations

For tolerance-related mutations, epegRNAs (Supplemental Table 6) were designed to induce single or double mutations using the ePE2 system (Li *et al.*, 2023). Transgenic callus events were genotyped using the Hi-TOM assay. The callus of edited events was transferred to the medium supplemented with 6 mM glyphosate for herbicide identification.

Seeds from self-pollinated T_0 lines of Y248H, T466I, and M472T were germinated for heritability assays. The targeted mutations were confirmed in the seedlings by Sanger sequencing. Homozygous mutants of Y248H and M472T, and heterozygous mutants of T466I, were grown to the four-leaf stage in Kimura's B solution culture medium. The tolerances were quantitively examined by a 3WP-2000 laboratory automatic spray tower (Nanjing Institute of Agricultural Mechanization, Ministry of Agriculture and Rural Affairs, China).

Supplemental References:

Clement, K., Rees, H., Canver, M.C., Gehrke, J.M., Farouni, R., Hsu, J.Y., Cole, M.A., Liu, D.R., Joung, J.K., Bauer, D.E., et al. (2019). CRISPResso2 provides accurate and rapid genome editing sequence analysis. Nature Biotechnology **37**:224-226. 10.1038/s41587-019-0032-3.

Hao, L., Ruiying, Q., Xiaoshuang, L., Shengxiang, L., Rongfang, X., Jianbo, Y., and Pengcheng, W. (2019). CRISPR/Cas9-Mediated Adenine Base Editing in Rice Genome. Rice Science **26**:125-128. https://doi.org/10.1016/j.rsci.2018.07.002.

Hu, L., Li, H., Qin, R., Xu, R., Li, J., Li, L., Wei, P., and Yang, J. (2016). Plant phosphomannose isomerase as a selectable marker for rice transformation. Scientific Reports **6**:25921. 10.1038/srep25921.

Jiang, Y., Xiao, Z., Luo, Z., Zhou, S., Tong, C., Jin, S., Liu, X., Qin, R., Xu, R., Pan, L., et al. (2025). Improving Plant C-to-G Base Editors with A Cold-adapted Glycosylase and TadA-8e Variants. Trends in biotechnology **Accepted**.

Li, J., Xu, R., Qin, R., Liu, X., Kong, F., and Wei, P. (2021). Genome editing mediated by SpCas9 variants with broad non-canonical PAM compatibility in plants. Molecular Plant **14**:352-360. 10.1016/j.molp.2020.12.017.

Li, J., Ding, J., Zhu, J., Xu, R., Gu, D., Liu, X., Liang, J., Qiu, C., Wang, H., and Li, M. (2023). Prime editing-mediated precise knockin of protein tag sequences in the rice genome. Plant communications **4**.

Liu, X., Gu, D., Zhang, Y., Jiang, Y., Xiao, Z., Xu, R., Qin, R., Li, J., and Wei, P. (2024). Conditional knockdown of OsMLH1 to improve plant prime editing systems without disturbing fertility in rice. Genome Biology **25**:131. 10.1186/s13059-024-03282-y.

Neugebauer, M.E., Hsu, A., Arbab, M., Krasnow, N.A., McElroy, A.N., Pandey, S., Doman, J.L., Huang, T.P., Raguram, A., Banskota, S., et al. (2023). Evolution of an adenine base editor into a small, efficient cytosine base editor with low off-target activity. Nature Biotechnology **41**:673-685. 10.1038/s41587-022-01533-6.

Sun, T., Liu, Q., Chen, X., Hu, F., and Wang, K. (2024). Hi-TOM 2.0: an improved platform for high-throughput mutation detection. Science China Life Sciences:1-3.

Xu, R., Liu, X., Li, J., Qin, R., and Wei, P. (2021). Identification of herbicide resistance OsACC1 mutations via in planta prime-editing-library screening in rice. Nature Plants **7**:888-892. 10.1038/s41477-021-00942-w.

Zhang, A., Shan, T., Sun, Y., Chen, Z., Hu, J., Hu, Z., Ming, Z., Zhu, Z., Li, X., He, J., et al. (2023). Directed evolution rice genes with randomly multiplexed sgRNAs assembly of base editors. Plant Biotechnology Journal **21**:2597-2610. https://doi.org/10.1111/pbi.14156.

Zhou, Q., Gao, Q., Gao, Y., Zhang, Y., Chen, Y., Li, M., Wei, P., and Yue, Z. (2024). BES-Designer: A Web Tool to Design Guide RNAs for Base Editing to Simplify Library. Interdisciplinary Sciences: Computational Life Sciences 10.1007/s12539-024-00663-6.

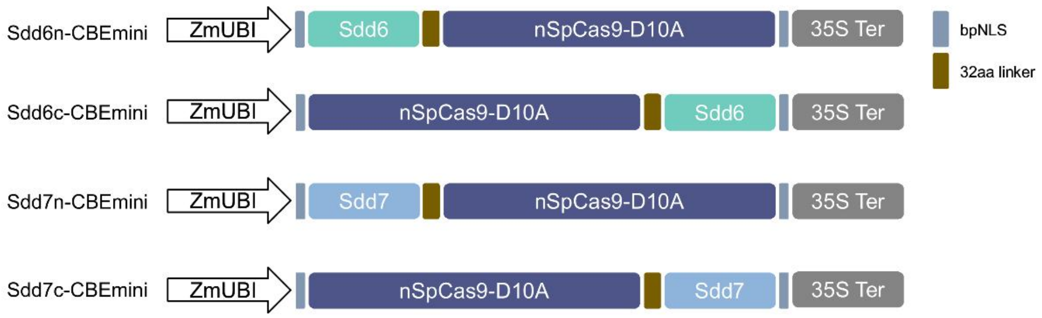

Supplemental Figure 1. Expression cassettes of the Sdd-derived CBEminis.

Schematic illustrations of the engineered CBEminis. Sdd6 or Sdd7 were fused to the N- or C-terminus of the SpCas9 D10A nickase with a 32 aa linker. The bipartite nuclear localization signal (bpNLS) was fused at both the N- and C-termini. ZmUBI, maize ubiquitin 1 promoter; 35S Ter, CaMV35S terminator.

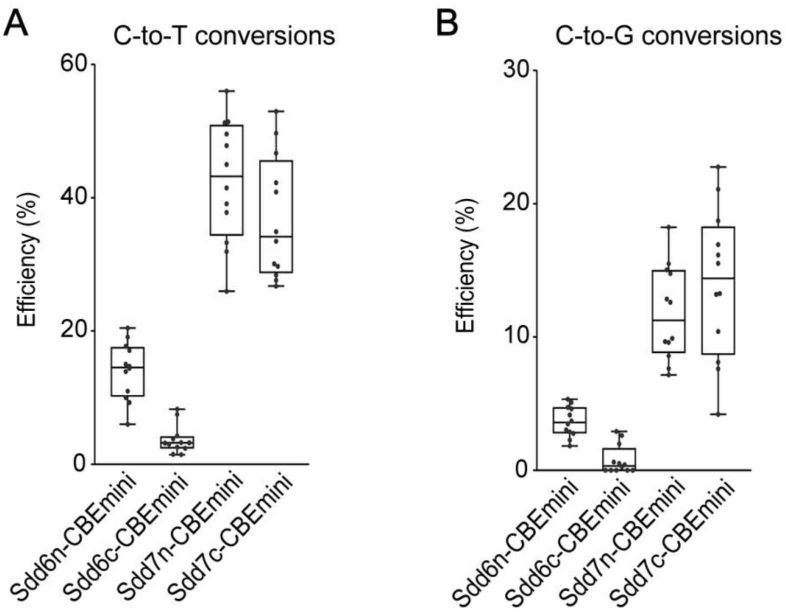

Supplemental Figure 2. Cytosine conversion efficiencies of the CBEminis.

The efficiency was calculated as the ratio of the number of C-to-T (A) or C-to-G (B) reads to the number of total clean reads. Box plot indicated the conversions at four endogenous targets with three biological replicates.

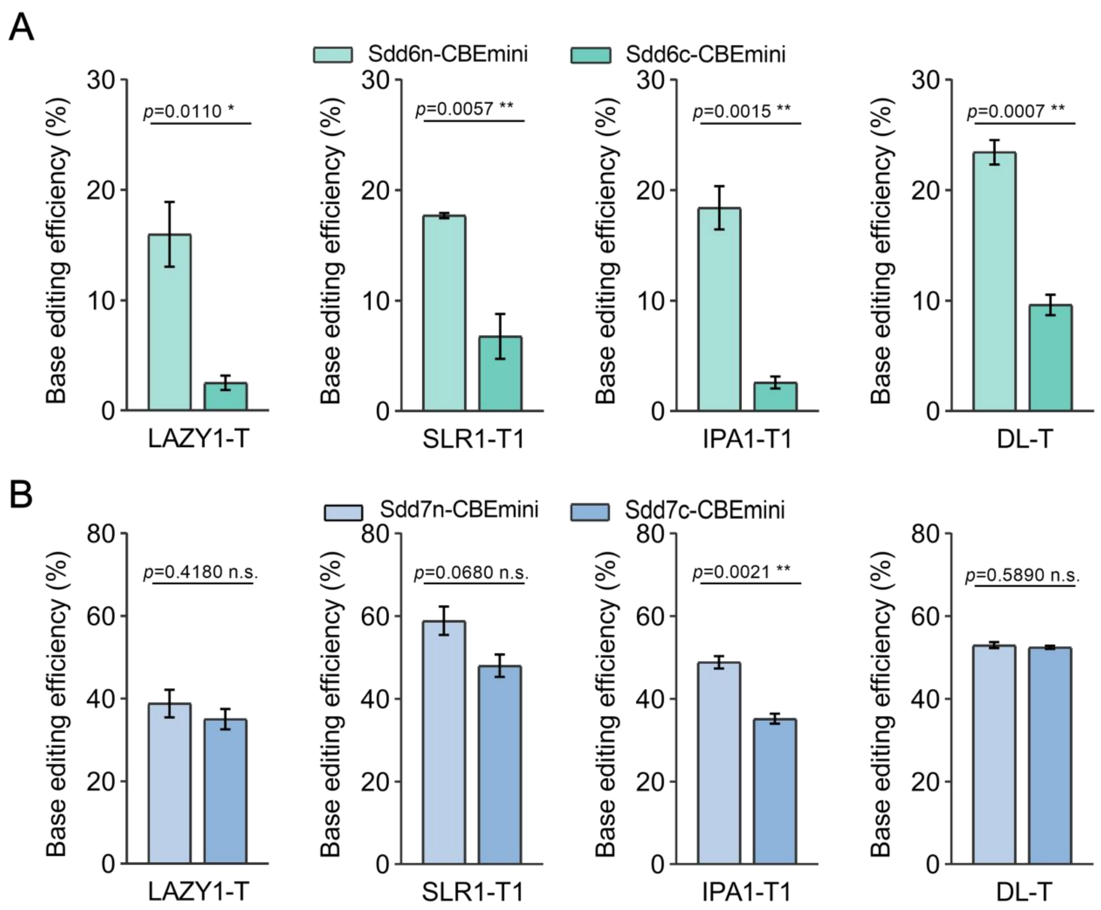

Supplemental Figure 3. Base editing efficiency of Sdd-CBEminis in stably transformed rice cells.

Paired comparisons of N- or C-terminal fused Sdd6 (A) or Sdd7 (B) in the CBEmini architecture at four individual targets. The efficiency was calculated as the ratio of the number of base edited reads to the number of total clean reads. The mean values were generated from three biological replicates. The newly emerged calli from approximately 200 independent events of a transformant were used to determine the efficiency in the cell population. Differences were determined by two-tailed *t* tests. *, *P*<0.05; **, *P*<0.01. n.s., not significant.

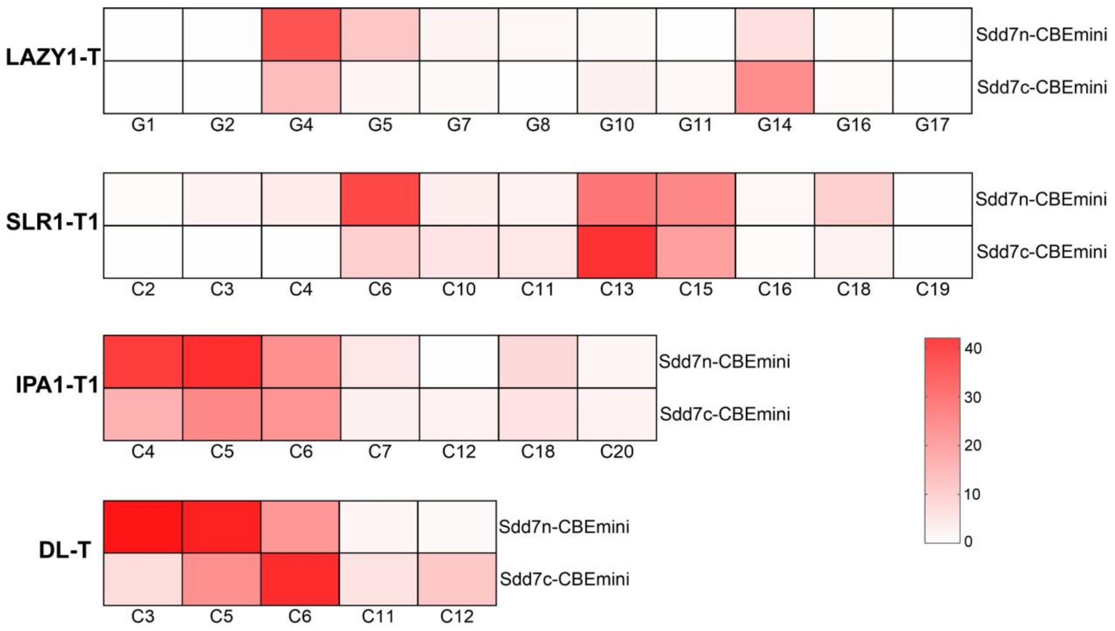

Supplemental Figure 4. Heatmap of base editing efficiencies induced by Sdd7n- and Sdd7c-CBEmini in calli.

The conversion efficiencies of editable C or G in the protospacer region are presented for each target. The position was counted from the first nucleotide of the protospacer distal to the PAM. The mean ratios of edited reads of the specific nucleotide to total clean reads were calculated from three biological replicates.

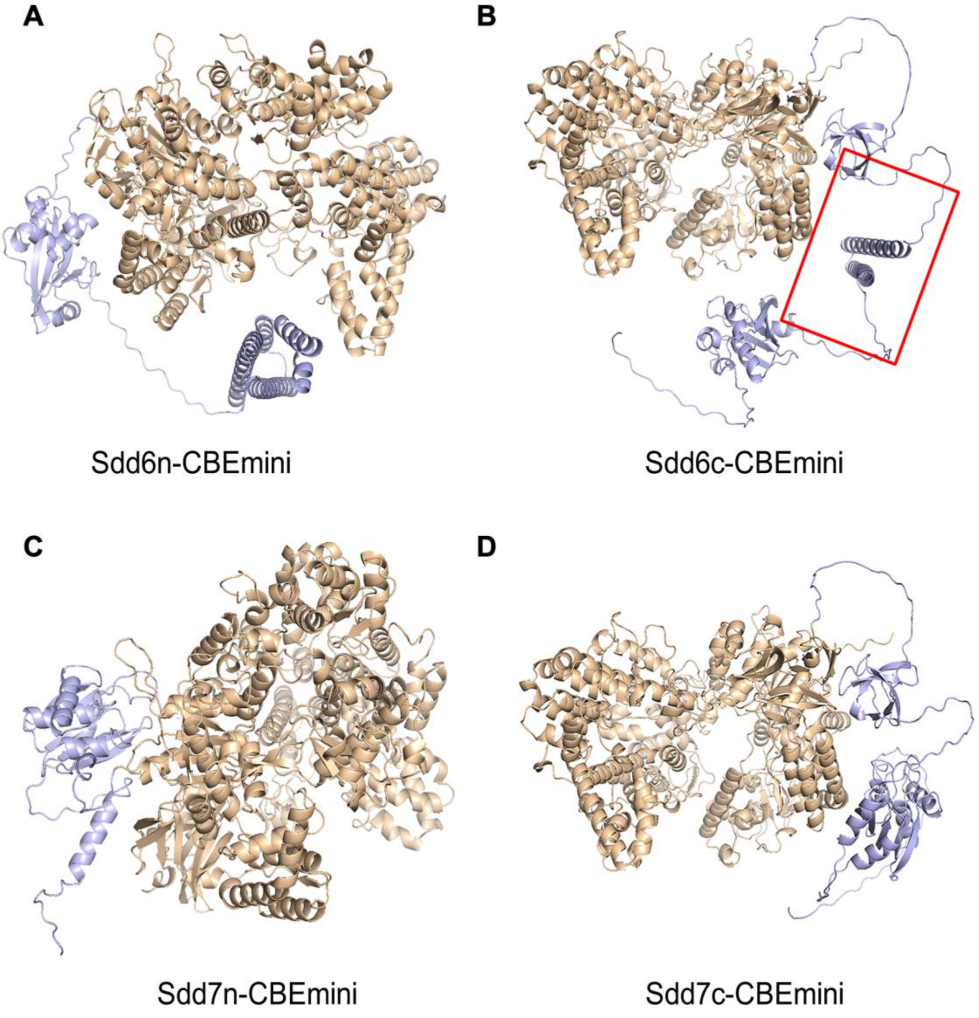

Supplemental Figure 5. Three-dimensional protein structures of the Sdd-CBEminis.

Protein complex models of Sdd6n-CBEmini (A), Sdd6c-CBEmini (B), Sdd7n-CBEmini (C), and Sdd7c-CBEmini (D) were predicted with AlphaFold3 with average per-residue confidence metric predicted local-distance difference test (pLDDT) ≥ 70. Then protein structure diagrams were visualized with PyMOL (version:3.1.6.1). The sequence of Sdd deaminase and nCas9 were highlighted by blue and yellow, respectively. The disordered domains of Sdd6 in the Sdd6c-CBEmini were indicated with a red box.

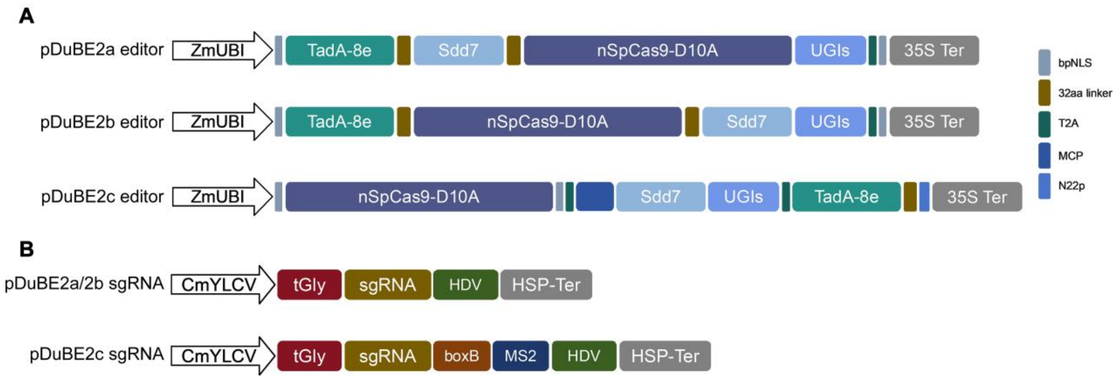

Supplemental Figure 6. Expression cassette structures of Sdd7-derived dual base editors.

A, Illustrations of pDuBE2 fusions. Sdd7, TadA-8e and triplet copies of Uracil-DNA glycosylase inhibitors (UGIs) were assembled into the SpCas9 D10A nickase with a 32 aa linker for pDuBE2a and pDuBE2b. In pDuBE2c, MS2 coat protein (MCP) and λ_{N22} protein (N22p) were fused to Sdd7 and TadA-8e, respectively, and assembled into nSpCas9 with a T2A self-cleavage peptide. The bipartite nuclear localization signal (bpNLS) was fused at both the N- and C-termini of nSpCas9, while SV40 NLSs were attached to the deaminases. All sequences were codon optimized for rice expression. B, sgRNA expression cassette. The OsU3 promoter was used for sgRNA expression in pDuBE2a and pDuBE2b. A Pol II promoter CmYLCV expressed sgRNA with tRNA-Gly (tGly) and hepatitis delta virus (HDV) ribozymes in pDuBE2c. MS2 and boxB aptamer RNAs were attached to the 3’ end of the sgRNA for the MoBE system. HSP-Ter, terminator of *AtHSP18.2*.

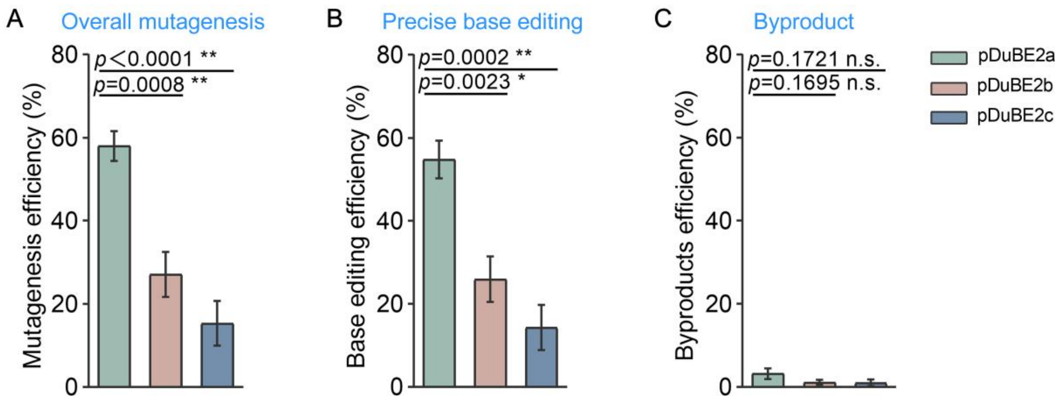

Supplemental Figure 7. Efficiency of pDuBE2s editing outcomes in rice calli.

The reads harboring any mutation at targets, A-to-G and/or C-to-T base conversions, and Indels or non-desired conversions were used to calculate overall mutagenesis efficiency (A), base editing efficiency (B), and byproducts efficiency (C), respectively. The read that simultaneously harboring base editing and indels was counted as byproduct edits. Mean value and standard variations of outcome efficiency at the six sites were presented. One-way ANOVA was applied to compare the difference of pDuBE2 efficiencies. *, *P*<0.05. n.s., not significant.

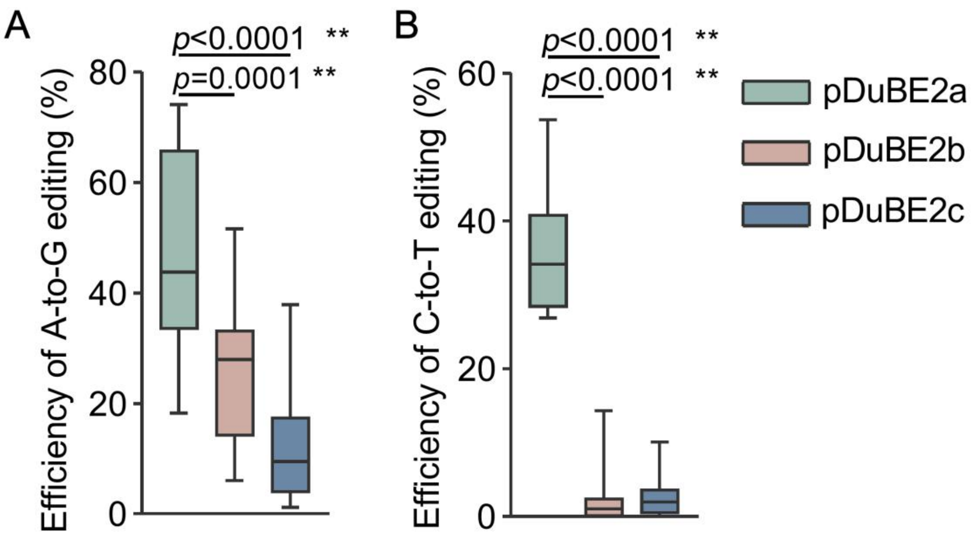

Supplemental Figure 8. Base editing profile of the pDuBE2s in rice calli.

The reads carrying A-to-G (A) or C-to-T (B) conversions were accounted for to calculate the efficiencies. The A&CBE edits of concurrent A-to-G and C-to-T conversions were applied to calculate both the A-to-G and C-to-T conversion efficiencies. The overall profiles of each pDuBE2 are presented in a box plot of base conversions at the six sites with three biological replicates. One-way ANOVA was applied to compare the difference of pDuBE2 efficiencies. **, P<0.01.

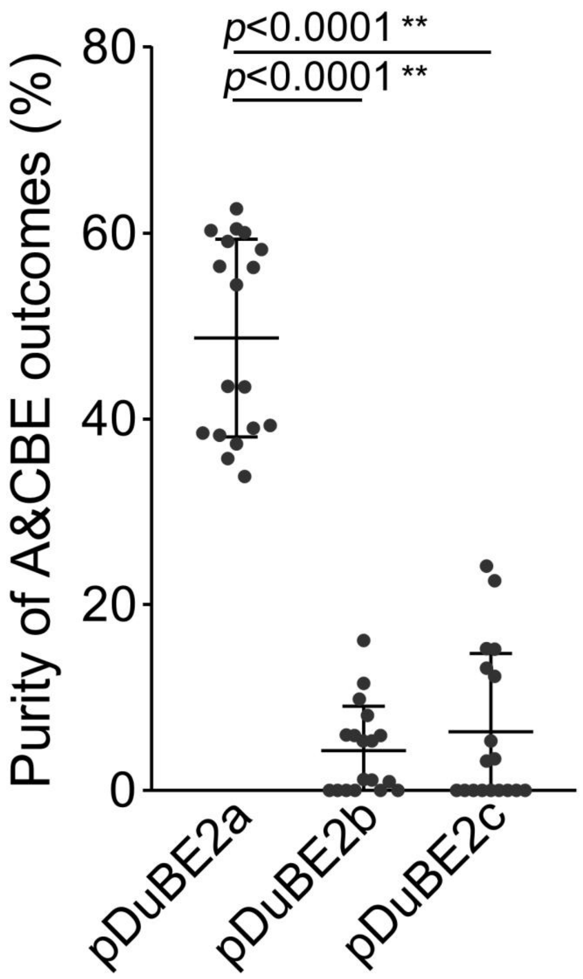

Supplemental Figure 9. A&CBE purity of pDuBE2s outcomes in rice calli.

The A&CBE outcome purities are presented as dots in a box plot (n=18), which were calculated as the ratio of simultaneous A-to-G & C-to-T edits reads to base-edited reads. The purities were determined at the six sites in the callus populations. The mean values and standard derivations are presented. The differences were assessed by the One-way ANOVA tests. **, *P*<0.01.

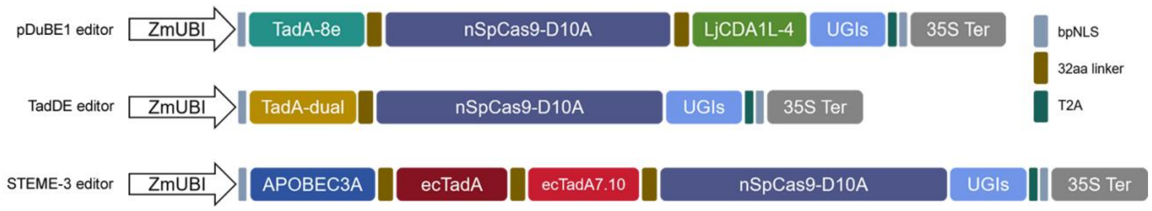

Supplemental Figure 10. Illustrations of the established plant dual base editors for the efficiency comparison with pDuBE2a.

The fusions of pDuBE1, TadDE, and STEME-3 were assembled according to the previous reports. The sequences of nSpCas9, TadA, and UGIs of the editors were optimized in the same manner with pDuBE2a. To strictly compare activities, the fusions were inserted into the plasmid backbone to replace the pDuBE2a editor. UGIs, triplet copies of UGI separating with T2A linkers. The OsU3 promoter-derived sgRNA expression cassette was applied for three editors.

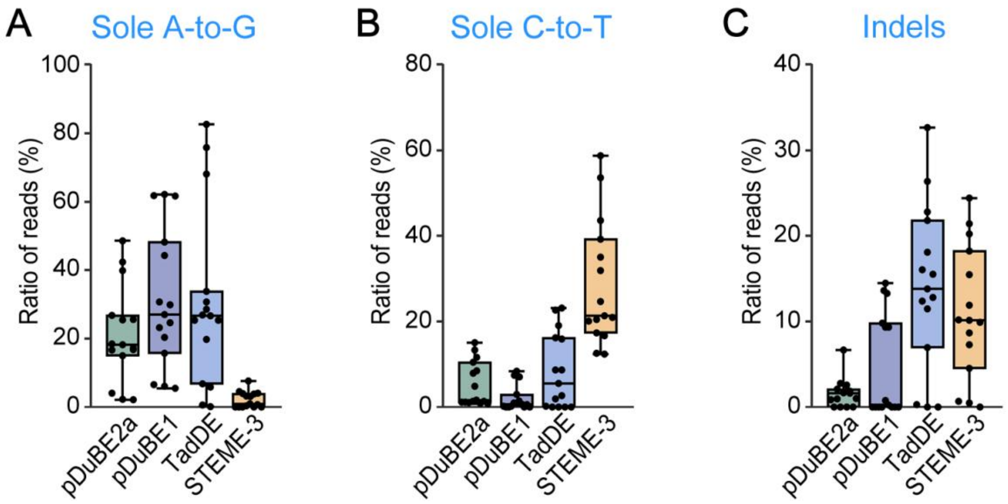

Supplemental Figure 11. Efficiencies of non-A&CBE outcomes of the plant dual base editors in rice calli.

The editing outcomes of pDuBE2a, pDuBE1, TadDE, and STEME-3 were compared by amplicon NGS in rice calli. The ratio of sole A-to-G (A), sole C-to-T (B), and indels (C) reads to total clean reads are indicated. The mean values and standard deviations of each plant dual base editor were generated from the five targets with three replicates and are presented in the box plots.

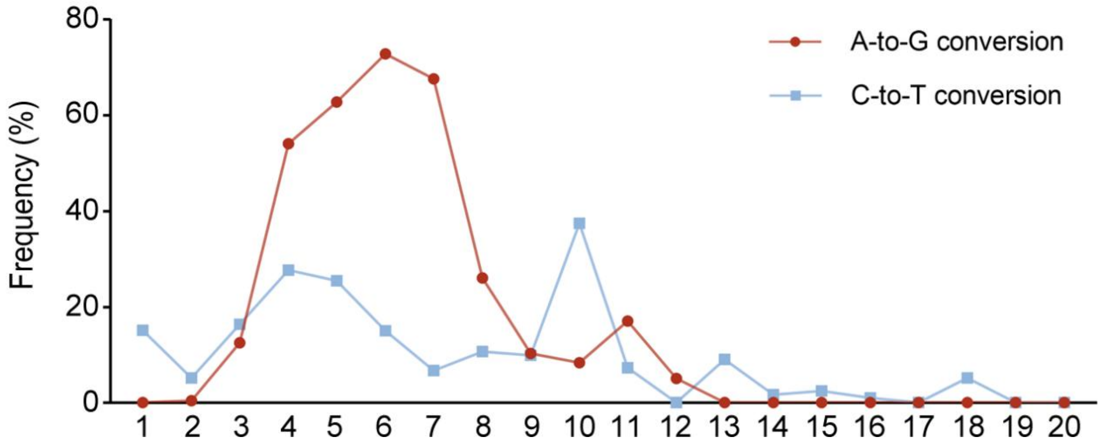

Supplemental Figure 12. ABE and CBE editing frequency of pDuBE2a in transgenic rice plants.

The editing frequency at individual nucleotides of the protospacer was calculated by the ratio of lines harboring the targeted substitution to the examined lines. The mean values of the A-to-G/C-to-T conversion frequencies were averaged from the ten genomic targets. The position was counted from the first nucleotide of the protospacer distal to the PAM.

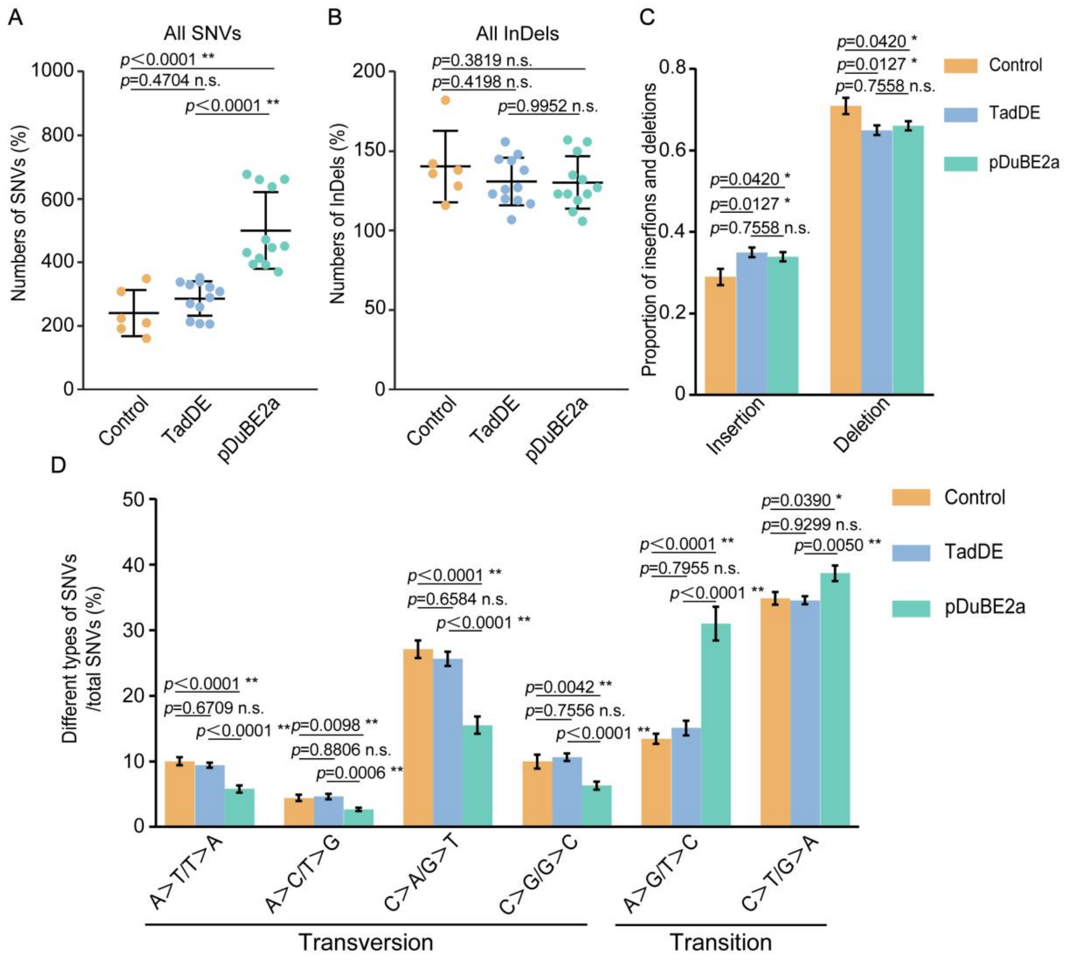

Supplemental Figure 13. Genome-wide off-target assessments of pDuBE2a in T_0 transgenic rice.

The whole genome of six SpCas9 plants (control group), twelve TadDE plants and twelve pDuBE2a plants were sequenced for at least 50-fold. Nine wild-type seedlings were simultaneously sequenced to filter the background mutations. A, Number of SNVs in the transgenic plants. The number of SNVs in each individual line is indicated by a dot. The numbers of SNVs in pDuBE2a plants were compared with those in the SpCas9 backbone control plants and TadDE lines. B, Number of InDels in the transgenic plants. C, Proportions of insertions and deletions among InDels identified in the transgenic plants. D, Comparison of different types of SNVs in the transgenic

plants. The means ± SDs are presented. The differences were assessed by a two-sided Mann–Whitney test. *, $P<0.05$; **, $P<0.01$. n.s., not significant.

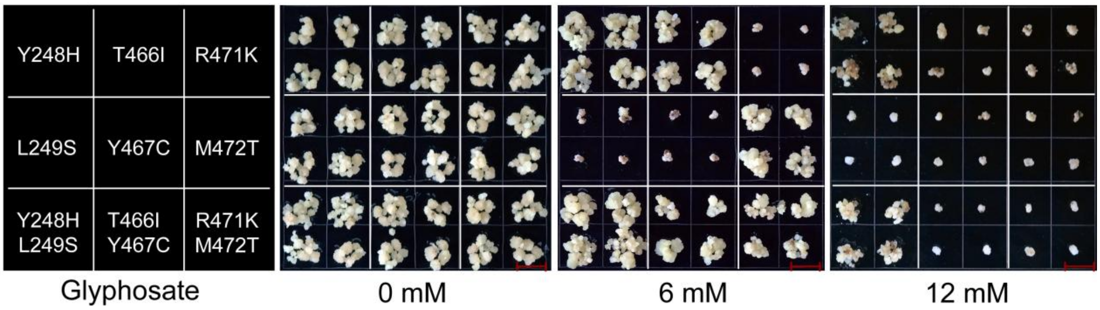

Supplemental Figure 14. Glyphosate tolerance of rice calli harboring OsEPSPS mutations.

The screened mutations of *OsEPSPS* were reproduced by the ePE2 system for precise editing (the left plate). After transformation, the infected calli were selected with hygromycin for 3 weeks. The prime editing was verified by sequencing in independent transformation calli. The edited calli were subsequently retransformed to new medium for the evaluation of herbicide tolerance. For each mutation, the calli were selected from the same edited event. The medium supplemented with 6 mM or 12 mM glyphosate were applied to the edited calli for 4 weeks. The calli were also grown on medium without glyphosate for 2 weeks as an internal control. Scale bar = 1 cm.

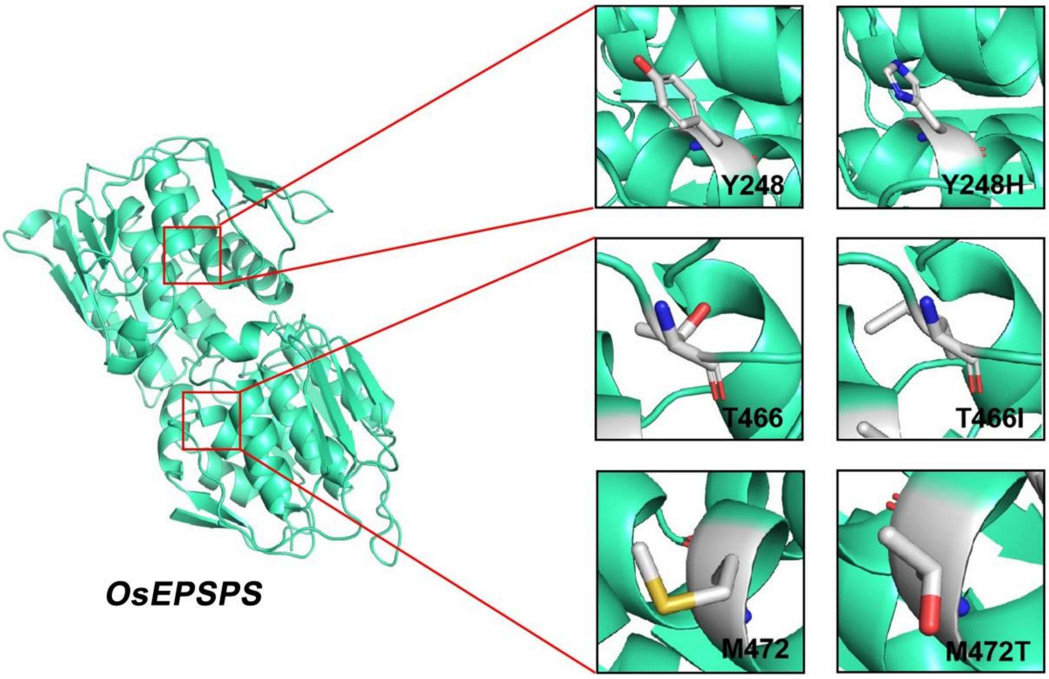

Supplemental Figure 15. Structural model of the OsEPSPS and molecular interactions with glyphosate.

The interactions between glyphosate and the residues were predicted. The wild-type residues (left) and the Y248H, T466I, and M472T mutations (right) are shown. Structural models of wild-type and mutant proteins were generated using AlphaFold3, followed by comparative visualization of conformational changes through residue substitution in PyMOL.

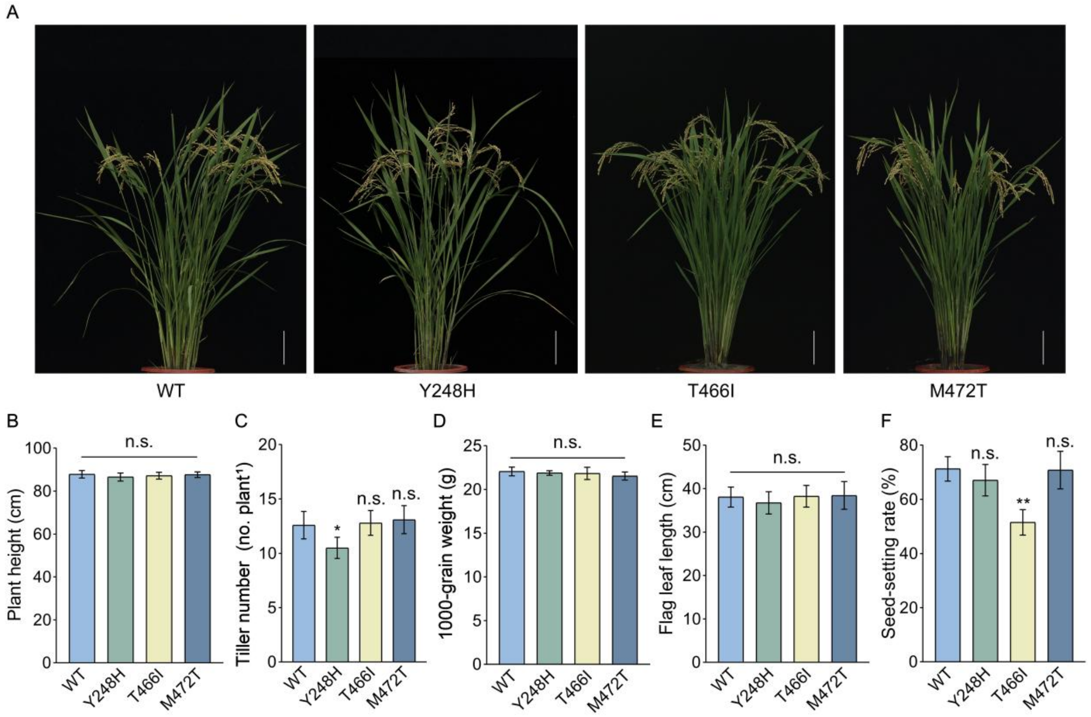

Supplemental Figure 16. Phenotypic analysis of screened OsEPSPS mutants.

The T-DNA-free T_1 progeny of homozygous Y248H and M472T mutants, along with heterozygous T466I mutants, were evaluated. All plants, including the wild-type Nipponbare control (WT), were cultivated in a field of Hefei during the summer of 2025 without treatment of glyphosate. The plants were pictured after 4 months growth (A). Partial sterile was observed in T466I mutants. Scale bar= 10 cm. The agronomic traits, including height (B), tiller numbers (C), thousand grain weight (D), leaf length (E), and seed-setting rate (F) were analysis from ten independent plants of each mutation. The significance between the OsEPSPS variant to WT was determined by one-way ANOVA analysis. $P<0.05$, *; n.s., not significant.

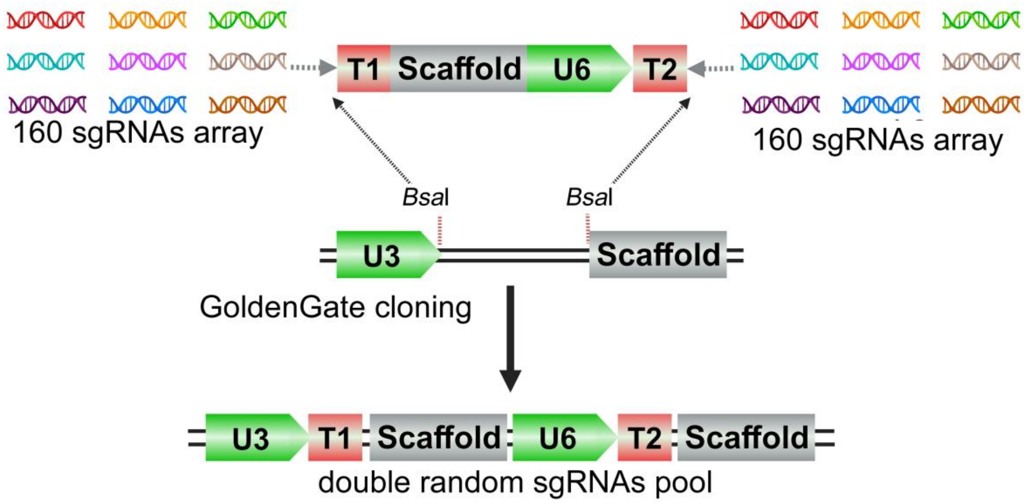

Supplemental Figure 17. Schematic illustration of double sgRNA array construction.

The OsU3 promoter (U3) and OsU6 promoter (U6) were used to simultaneously express a pair of sgRNAs in a pDuBE2a vector. The sgRNA arrays were separately synthesized with 3' adapters for the sgRNA scaffold sequence (T1 site) and with 5' adapters for the OsU6 sequence (T2 site). A random pair of gRNAs was then integrated into the dual expression cassette by amplifying the scaffold-OsU6 chimeric sequence. The pooled amplicons were assembled downstream of the OsU3 promoter in pDuBE2a through the Goldengate strategy, resulting in a double sgRNA library.

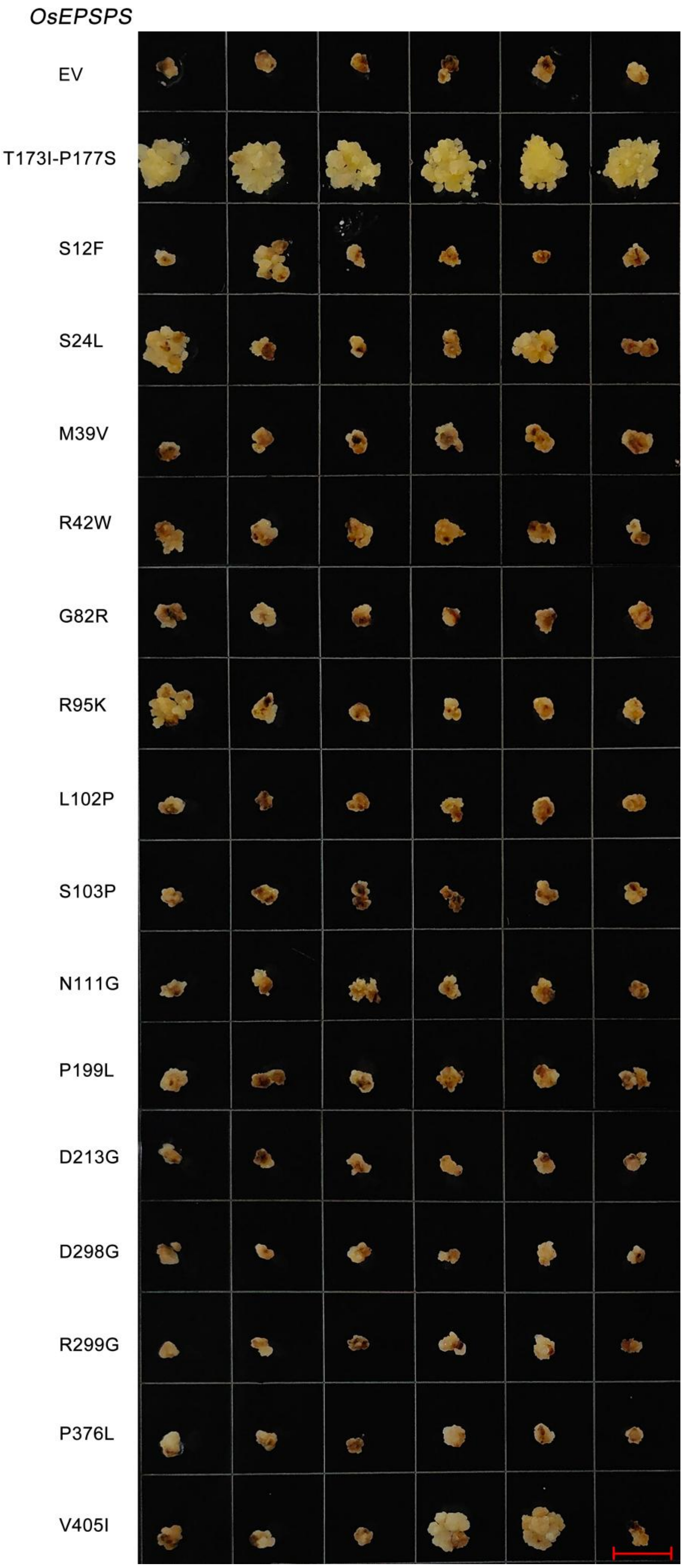

OsEPSPS
EV
T173I-P177S
S12F
S24L
M39V
R42W
G82R
R95K
L102P
S103P
N111G
P199L
D213G
D298G
R299G
P376L
V405I

Supplemental Figure 18. Glyphosate tolerance identification of the mutations disassembled from double sgRNA library-screened complex edits.

The complex edits screened from the double sgRNA library (Supplemental Table 4) were disassembled into single mutations. The 15 types of novel single mutations were then obtained point-by-point through prime editing and genotyped in calli. For each mutation, 6 edited calli were transformed for selection under 6 mM glyphosate for 4 weeks. The T173I-P177S mutation in OsEPSPS, a well-characterized modification known to confer glyphosate tolerance in rice, were used as positive controls, and the calli transformed with the empty ePE2 vector (EV) were used as negative controls. Scale bar = 1 cm.

Supplemental Table 1. Base editing of pDuBE2a in T_0 transgenic rice plants.

Targets	Tested lines	Base editing lines*	Simultaneous A/C conversions&	ABE lines#	CBE lines#	A&CBE lines#	
						Total	Ho@
ALS-T1	48	20	15 (31.25%)	7	11	15 (31.25%)	3
IPA1-T2	48	36	30 (62.50%)	24	0	30 (62.50%)	6
PDS-T1	48	34	31 (64.58%)	7	0	31 (64.58%)	9
PDS-T2	48	24	15 (31.25%)	25	4	12 (25.00%)	2
PDS-T3	48	40	29 (60.42%)	34	24	16 (33.33%)	0
Pikh-T1	48	22	20 (41.67%)	0	4	20 (41.67%)	6
Pikh-T2	48	37	29 (60.42%)	3	23	28 (58.33%)	10
SLR1-T2	48	34	26 (54.17%)	16	12	26 (54.17%)	2
SLR1-T3	48	34	27 (56.25%)	8	0	27 (56.25%)	5
WX-T	48	24	16 (33.33%)	4	4	16 (33.33%)	1

*, number of lines harboring base conversions in the protospacer region;

&, number of lines harboring simultaneous A-to-G and C-to-T conversions; the A-to-G edit and C-to-T edit could be separate alleles of one transgenic plant; the ratio of simultaneous A/C conversion lines was calculated in brackets;

#, number of lines harboring an editing allele of sole A-to-G conversion (ABE outcome), an editing allele of sole C-to-T conversion (CBE outcome), and an editing allele of concurrent A-to-G and C-to-T conversion (A&CBE outcome); a T_0 line could be a chimeric plant of one or more edits; the ratio of plants harboring A&CBE outcomes was calculated in brackets. Please note that A&CBE lines are included within the category of simultaneous A/C conversions.

@, number of lines harboring homozygous A&CBE edits.

Supplemental Table 2. Transmission of edits and T-DNA in the T_1 generation of pDuBE2a.

Target	No. of T_0*	T_0 Zygosity^	Number of T_1 plants	Segregation of edits#	T-DNA&
ALS-T1	#15	Ho	18	18Ho	16+:2-
	#27	Ho	12	12Ho	12+
	#3	He	15	7Ho:6He:2WTs	12+:3-
PDS-T1	#6	Ho	15	15Ho	14+:1-
	#11	Ho	13	13Ho	10+:3-
	#2	Chi	30	9Ho: 2Bi: 17HE: 2WT	25+:5-
Pikh-T2	#1	Ho	20	20Ho	19+:1-
	#7	Ho	15	15Ho	11+:4-
	#6	Chi	22	3Ho: 12He: 7WT	22+

*, Self-crossing seeds of nine T_0 pDuBE2a-edited lines at the ALS-T1, PDS-T1 and Pikh-T_2 target sites were used for progeny genotyping. For each target, two homozygous and one heterozygous/chimeric T_0 mutant were selected;

^, the zygosity of selected T_0 lines were indicated. Ho, homozygous mutant; Chi, Chimeric mutant;

#, The targeted edits were assessed by Hi-TOM analysis with a 15% threshold. All edits obtained from T_1 plants have been occurred in their corresponding T_0 parent. He, heterozygous mutant; WT, wild type plant.

&, Two pairs of primers were designed to detect T-DNA segregation in the T_1 generation. The numbers of T-DNA-positive (+) and T-DNA-negative (-) plants are indicated.

Supplemental Table 3. Inheritance of the point mutations in rice.

Mutations	T_0 line	T_0 zygosity*	T_1 progenies#		
			Total	Ho	He
Y248H	No.4	He	24	9	11
	No.15	Ho	16	16	0
T466I	No.2	He	48	0	28
	No.3	He	48	0	12
	No.7	He	20	0	7
	No.12	He	24	0	5
M472T	No.1	Ho	24	24	0
	No.4	Ho	24	24	0

*, the point mutations were edited by the ePE2 system. Zygosity was identified by a Hi-TOM assay with a 15% threshold. T_0 lines with homozygous (Ho) and/or heterozygous (He) mutations were self-pollinated to produce offspring. The homozygous T466I mutation was absent in the T_0 generation.

#, The seeds of the T_0 lines were randomly selected for germination. T_1 seedlings were genotyped.

Supplemental Table 4. Glyphosate-resistant *OsEPSPS* mutations screened by the pDuBE2a library with the double-sgRNA array.

Mutations*	Edits&	Types of editing#
S24L-Y248H	TCG/TAC-to-TTG/CAC	A&CBE
R95K-Y248H	AGG/TAC-to-AAG/CAC	A&CBE
D213G-Y248H	GAT/TAC-to-GGT/CAC	ABE
M39V-Y248H-L249S	ATG/TAC/TTG-to-GTG/CAC/TCG	ABE
G82R-Y248H-L249S	GGG/TAC/TTG-to-AGG/CAC/TCA	A&CBE
Y248H-L249S-P376L	TAC/TTG/CCA-to-CAC/TCA/TCA	A&CBE
S12F-M279T	TCC/ATG-to-TTC/ACG	A&CBE
N111G-M279T	AAC/ATG-to-GGC/ACA	A&CBE
S12F-E278K-M279T	TCC/GAA/ATG-to-TTT/AAA/ACG	A&CBE
P199L-T466I	CCG/ACC-to-TTG/ATT	CBE
L102P-S103P-T466I	CTC/TCC/ACC-to-CCC/CCC/ATC	A&CBE
V405I-T466I-Y467C	GTT/ACC/TAC-to-ATT/ATC/TGC	A&CBE
R42W-M472T	CGG/ATG-to-TGG/ACG	A&CBE
D298G-R299G-M472T	GAC/AGA/ATG-to-GGC/GGA/ACG	ABE

*, new mutations of an edited allele identified by Sanger sequencing of a PCR clone of the *OsEPSPS* gene in herbicide-resistant callus events; the gray box indicates new mutations screened by the double sgRNA array;

&, triplets of the missense mutations; the edited nucleotides are labeled in red;

#, Types of pDuBE2a activity for conducting mutations.

Supplemental Table 5. DuBE-editing related oligos in the study.

1. Protospacer sequences used for base editing.

Target	Sequence (5' to 3')
ALS-T1	GCTGCCTATGATCCCAAGTGGGG
BADH2-T	ATCACACCCTGGTGTAGACAAGG
DL-T	GACACCGTGACCGTGAAATGTGG
IPA1-T1	AAGCCCCTGGACAGGTTCTCGGG
IPA1-T2	CCGCCACCGACTCGAGCTGTGCT
LAZY1-T	CCCCGCGGCGACGGCGGCGGAGG
PDS-T1	CCATGTTCGCTCTTTGGGTGGTG
PDS-T2	CCACTAAACCATTACAGGTCGTG
PDS-T3	AATGCTAACTTGGCCAGAGAAGG
Pikh-T1	CCATGTCTGAAGATTGTGGAGAT
Pikh-T2	AGCTCGAAGGAAGGTCTGAGAGG
SLR1-T1	CCCCTCGGACCTCTCCTCCTGGG
SLR1-T2	CTCCACCACAGAGCCCCGCGAGG
SLR1-T3	CCTCGGACCTCTCCTCCTGGGTC
WX-T	GACGCCCAAGCAGCAGCGGTCGG

The PAM sequence is underlined.

2. Primers for amplicon-NGS.

Target	Sequence (5' to 3')
ALS-T1	FP: AAGTCCGTGCCGCCATCAAGAA<br>RP: GGCTGGTGCTTTGCCAACATAC
BADH2-T	FP: GTTGGTCTTCCTTCAGGTGTGC<br>RP: ATAACTCCCAGTAAATGCAACC
DL-T	FP: TCTTTTGGGTAGCTGCAGGT<br>RP: CAAGGGGTGATCAGTTGGGG
IPA1-T1	FP: TCCACTACCACCAGCTTTGA<br>RP: TTCATGTGGTAGCTGGTGCG
IPA1-T2	FP: GTGTTCGCTGGCCCAAATCT<br>RP: GACATGGCTGCAGCCTGGTT
LAZY1-T	FP: TCGACTTCGAGGTGGACGCCA<br>RP: CGTCCTCCGTGGTGGTGTCGT
PDS-T1	FP: AGGAGAAGCATGGTTCTAAG<br>RP: GTTCCATCAGTAAGTGCAAA
PDS-T2	FP: GCCAGGACTTTCCAAGACCTCC<br>RP: GACCACGATGTGACTGCTATCA
PDS-T3	FP: TATGTTGTCAATTGATGCCTAT<br>RP: TCATCCACTCAGAAACAGTAAA
Pikh-T1	FP: CTCACATCTCTGGTTGAGAT<br>RP: ACTGATATTCTCAACTCGGC
Pikh-T2	FP: TTGAGAGCACTGCATATGGAGT<br>RP: GGCGATCAAGAGCCTGTAGACC
SLR1-T1	FP: GGATGACGGGTTCGTGTCGC<br>RP: CTGGGAGTTCAAAGAAGCCG
SLR1-T2	FP: ACACCAAGCGGATGCGCACT<br>RP: GCGTGTCAACCACCACAACC
SLR1-T3	FP: CCATGGGGATGGGCGGCGTGAG<br>RP: CGCCGGTGACAGTGGACGAGGT
WX-T	FP: TTCCAGGGCCTCAAGCCCCGCA<br>RP: GGCGCCGACGAACACGACGTTC
sgRNA array	FP: GTATGGGCCGGCCCATTACG<br>RP: TAGACATGCAATGCTCATTA

3. Primers for Hi-TOM analysis.

Target	Sequence (5' to 3')
ALS-T1	FP: ggagtgagtacggtgtgcGTGCCGCCATCAAGAAGATG<br>RP: gagttggatgctggatggGGTGCTTTGCCAACATACAG
IPA1-T2	FP: ggagtgagtacggtgtgcGTGTTCGCTGGCCCAAATCT<br>RP: gagttggatgctggatggGACATGGCTGCAGCCTGGTT
PDS-T1	FP: ggagtgagtacggtgtgcAGGAGAAGCATGGTTCTAAG<br>RP: gagttggatgctggatggGTTCCATCAGTAAGTGCAAA
PDS-T2	FP: ggagtgagtacggtgtgcGGACTTTCCAAGACCTCCAC<br>RP: gagttggatgctggatggCCACGATGTGACTGCTATCA
PDS-T3	FP: ggagtgagtacggtgtgcATGTTGTCAATTGATGCCTA<br>RP: gagttggatgctggatggTTCATCCACTCAGAAACAGT
Pikh-T1	FP: ggagtgagtacggtgtgcCTCACATCTCTGGTTGAGAT<br>RP: gagttggatgctggatggACTGATATTCTCAACTCGGC
Pikh-T2	FP: ggagtgagtacggtgtgcGAGAGCACTGCATATGGAGT<br>RP: gagttggatgctggatggCTGTAGACCCTCACAGCCTG
SLR1-T2	FP: ggagtgagtacggtgtgcACACCAAGCGGATGCGCACT<br>RP: gagttggatgctggatggGCGTGTCAACCACCACAACC
SLR1-T3	FP: ggagtgagtacggtgtgcGCGCCGCGGATGACGGGTTC<br>RP: gagttggatgctggatggAGGTGGAAGCATGGCGGGCA
WX-T	FP: ggagtgagtacggtgtgcTTCCAGGGCCTCAAGCCCCG<br>RP: gagttggatgctggatggGGCGCCGACGAACACGACGT
Y248H-L249S	FP: ggagtgagtacggtgtgcACTGAATTACACTCAACAGG<br>RP: gagttggatgctggatggTGGAGATTAGTTTGTCAATG
E278K-M279T	FP: ggagtgagtacggtgtgcCTGATGGCTGCTCCTTTGGC<br>RP: gagttggatgctggatggCTTGTACTTCTGCCCTCCCT
T466I-Y467C	FP: ggagtgagtacggtgtgcCTGGGAGCATCGGTTGAAGA<br>RP: gagttggatgctggatggTGCAACCAGGGTCCCTGATC
R471K-M472T	FP: ggagtgagtacggtgtgcCCTGACTACTGCATCATCAC<br>RP: gagttggatgctggatggCAGTTCCTGACGAAAGTGCT
S12F/S24L	FP: ggagtgagtacggtgtgcCAACCCCACCCCATCCTCCC<br>RP: gagttggatgctggatggGGCAGCCGCAGCTGCTTCCG
M39V/R42W	FP: ggagtgagtacggtgtgcGTCCCTGGACCAGGCCGTGG<br>RP: gagttggatgctggatggTGCCACCGACGACGACGACG
G82R/R95K/L102P/S103P	FP: ggagtgagtacggtgtgcCGTCGTCGTCGTCGGTGGCA<br>RP: gagttggatgctggatggCTCCAGAAATGGAATTCACG
N111G	FP: ggagtgagtacggtgtgcCTGATGCCTGCTTACATTGC<br>RP: gagttggatgctggatggCACAGAGAGCCCGAGGGCTT
P199L/D213G	FP: ggagtgagtacggtgtgcGTATGGAATTCATGGGGTAT<br>RP: gagttggatgctggatggCACGAACAGGTGGGCATTCA
D298G/R299G	FP: ggagtgagtacggtgtgcCTCCATTCCTTACGTTGAAA<br>RP: gagttggatgctggatggGTTAATCATAAACACCCGAA
P376L/V405I	FP: ggagtgagtacggtgtgcTACATGGACTGACACCAGTG<br>RP: gagttggatgctggatggGGCCTTAATGTTTACCATCT

4. Primers for Sanger sequencing.

Target	Sequence (5' to 3')
OsEPSPS gene	FP: ATGGCGTCCAACGCCGCGGCTG
	RP: TCAGTTCCTGACGAAAGTGCTT
OsEPSPS mutations	FP1: ATGGCGTCCAACGCCGCGGC
	FP2: ACGAATGAGGGAGAGACCGA
	FP3: ACTGCTTAGCATTGTGACAA
	RP1: CGACATCCGCACCAAGTTGT
	RP2: CCGTTCAGAAAATTGCCAGC
sgRNA	FP: GTATGGGCCGGCCCATTACG
	RP: TAGACATGCAATGCTCATTA

5. Primers for T-DNA segregations.

Target	Sequence (5' to 3')
CmYLCV-HSP-Ter	FP: GGCGAAGTATTCAGGCACGTGG
	RP: TCCATAGTCCATACCATAGCAC
T2A-35S Ter	FP: TACTCAAGCAAGCCGGCGATGT
	RP: GTTAGATCGACGTCGCATGCTC

Supplemental Table 6. Simplified sgRNA array of the *OsEPSPS* coding region.

No.	Target sequence	Strand	PAM
1	GCCGGCAATGGCGGCGACCA	+	TGG
2	GTCCAACGCCGCGGCTGCGG	+	CGG
3	GGACCAGGCCGTGGCGGCGT	+	CGG
4	GGGGGATGCGGGTGCGGGTG	+	CGG
5	GGTGGCAGCGCCGGCGGCGA	+	AGG
6	AGATCGTGCTCCAGCCCATC	+	AGG
7	GGTCCAAGTCGCTCTCCAAC	+	AGG
8	TTATTAGGGCACAACAGTGG	+	TGG
9	GGACAACTTGCTGAACAGTG	+	AGG
10	GGATGTTCACTACATGCTTG	+	AGG
11	CTTGAGGCCCTGAAAGCCCT	+	CGG
12	GCAAAAAGAGCTGTAGTCGT	+	TGG
13	GCTGTAGTCGTTGGCTGTGG	+	TGG
14	TGGCAAGTTTCCTGTTGAGA	+	AGG
15	TGTTGAGAAGGATGCGAAAG	+	AGG
16	AGAGGAAGTGCAACTCTTCT	+	TGG
17	ACAGCAGCCGTGACTGCTGC	+	TGG
18	ACCATCAGTTATGTGCTTGA	+	TGG
19	TTGATGGAGTGCCACGAATG	+	AGG
20	CGAATGAGGGAGAGACCGAT	+	TGG
21	GGAGAGACCGATTGGTGACT	+	TGG

22	GCGGATGTCGACTGTTTCCT	+	TGG
23	ATGCCCACCTGTTCGTGTCA	+	AGG
24	AAGGGAATTGGAGGACTTCC	+	TGG
25	TGGAGGACTTCCTGGTGGCA	+	AGG
26	GTACTTGAGTGCCTTGCTGA	+	TGG
27	CTTGCTGATGGCTGCTCCTT	+	TGG
28	TGAAATGACATTGAGATTGA	+	TGG
29	TTGAGATTGATGGAGCGTTT	+	TGG
30	GATGGAGCGTTTTGGTGTGA	+	AGG
31	CCTGGAAATGCCTATGTTGA	+	AGG
32	CTCAAGCGCGAGCTATTTCT	+	TGG
33	GGAGGCACTGTGACAGTTCA	+	AGG
34	ACTGTGACAGTTCAAGGTTG	+	TGG
35	TTGTGGTACGACCAGTTTGC	+	AGG
36	GGGTGATGTCAAATTTGCTG	+	AGG
37	ACTTGAGATGATGGGAGCAA	+	AGG
38	TGATGGGAGCAAAGGTTACA	+	TGG
39	GACACCAGTGTAACCGTAAC	+	TGG
40	GGTCCAACTGCTATCAGAGA	+	TGG
41	ATCAGAGATGGTAAACATTA	+	AGG
42	GGAGAGTAAAGGAAACCGAA	+	AGG
43	AGTAAAGGAAACCGAAAGGA	+	TGG
44	CCGAAAGGATGGTTGCAATT	+	CGG
45	AATTCGGACCGAGCTAACAA	+	AGG

46	CTAACAAAGGTAAATTCATT	+	AGG
47	CTGGGAGCATCGGTTGAAGA	+	AGG
48	CTACTGCATCATCACCCCAC	+	CGG
49	ACCGGAGAAGCTGAACATCA	+	CGG
50	TCGACACCTACGATGATCAC	+	AGG
51	CACCTACGATGATCACAGGA	+	TGG
52	CGATGATCACAGGATGGCCA	+	TGG
53	CCGACGTGCCCGTGACGATC	+	AGG
54	ACGTTCTAAGCACTTTCGTC	+	AGG
55	GCAATGGCGGCGACCATGGC	-	CCG
56	TGGCGTCCAACGCCGCGGCT	-	CCA
57	CGGCTGCGGCGGCGGTGTCC	-	CCG
58	GGCCGTGGCGGCGTCGGCGG	-	CCA
59	CGTCGTCGTCGTCGGTGGCA	-	CCG
60	GCCCATCAGGGAGATCTCCG	-	CCA
61	GGGCGGTTCAGCTGCCAGGG	-	CCG
62	GGGTCCAAGTCGCTCTCCAA	-	CCA
63	AGTCGCTCTCCAACAGGATC	-	CCA
64	CCTCCTCTCCGCCCTCTCCG	-	CCT
65	CCCTCTCCGAGGTGAGACGC	-	CCG
66	TGACTGCTGCTGGTGGAAAT	-	CCG
67	TCAGTTATGTGCTTGATGGA	-	CCA
68	CGAATGAGGGAGAGACCGAT	-	CCA
69	TGGCACTGAATGCCCACCTG	-	CCT

70	ACCTGTTCGTGTCAAGGGAA	-	CCC
71	GTTCGTGTCAAGGGAATTGG	-	CCT
72	TCAGCAGTCAGTACTTGAGT	-	CCA
73	TGCTGATGGCTGCTCCTTTG	-	CCT
74	TTGGCCCTTGGGGATGTGGA	-	CCT
75	TTGGGGATGTGGAGATCGAA	-	CCC
76	TACGTTGAAATGACATTGAG	-	CCT
77	GGAAATGCCTATGTTGAAGG	-	CCT
78	TAACTGGTCCACCACGTGAG	-	CCG
79	CCACGTGAGCCTTATGGGAA	-	CCA
80	TATGGGAAGAAACACCTGAA	-	CCT
81	GAAAGCTGTTGATGTCAACA	-	CCT
82	GATGTTGCCATGACCCTTGC	-	CCT
83	TGACCCTTGCCGTTGTTGCA	-	CCA
84	TTGCCGTTGTTGCACTCTTC	-	CCC
85	ACTGCTATCAGAGATGGTAA	-	CCA
86	CAGTGGCTTCCTGGAGAGTA	-	CCA
87	GGAGAGTAAAGGAAACCGAA	-	CCT
88	AAAGGATGGTTGCAATTCGG	-	CCG
89	AGCTAACAAAGGTAAATTCA	-	CCG
90	GCTGGGAGCATCGGTTGAAG	-	CCA
91	GACTACTGCATCATCACCCC	-	CCT
92	GAGAAGCTGAACATCACGGC	-	CCG
93	ACGATGATCACAGGATGGCC	-	CCT

94	TGGCCTTCTCCCTCGCTGCC	-	CCA
95	TCTCCCTCGCTGCCTGCGCC	-	CCT
96	ACGTGCCCGTGACGATCAGG	-	CCG
97	GTGACGATCAGGGACCCTGG	-	CCC
98	TGGTTGCACCCGCAAGACCT	-	CCC
99	GACCATGGCGTCCAACGCCG	+	CGG
100	GGCGTCCAACGCCGCGGCTG	+	CGG
101	CAACGCCGCGGCTGCGGCGG	+	CGG
102	GGCTGCGGCGGCGGTGTCCC	+	TGG
103	GGCGGCGGTGTCCCTGGACC	+	AGG
104	GGTGTCCCTGGACCAGGCCG	+	TGG
105	CCAGGCCGTGGCGGCGTCGG	+	CGG
106	CGTCGGCGGCGTTCTCGTCG	+	CGG
107	TCTCGTCGCGGAAGCAGCTG	+	CGG
108	GCAGCTGCGGCTGCCCGCCG	+	CGG
109	CGGCTGCCCGCCGCGGCGCG	+	CGG
110	CCGCGGCGCGCGGGGGGATG	+	CGG
111	TGCGGGTGCGGGTGCGGGCG	+	CGG
112	GGGTGCGGGTGCGGGCGCGG	+	GGG
113	GCGGGCGCGGGGGCGGCGGG	+	AGG
114	GGGGCGGCGGGAGGCGGTGG	+	TGG
115	GGCGTCCGCGTCGTCGTCGT	+	CGG
116	GTCCGCGTCGTCGTCGTCGG	+	TGG
117	GTCGTCGTCGGTGGCAGCGC	+	CGG

118	GTCGTCGGTGGCAGCGCCGG	+	CGG
119	GGCAGCGCCGGCGGCGAAGG	+	CGG
120	AGCGCCGGCGGCGAAGGCGG	+	AGG
121	CAGCCCATCAGGGAGATCTC	+	CGG
122	TCCGGGGCGGTTCAGCTGCC	+	AGG
123	GCCCTCTCCGAGGTGAGACG	+	CGG
124	GAAAGCCCTCGGGCTCTCTG	+	TGG
125	AGAGCTGTAGTCGTTGGCTG	+	TGG
126	CAACTCTTCTTGGGGAACGC	+	TGG
127	GCAGCCGTGACTGCTGCTGG	+	TGG
128	GTTGTCGGGTTGAAACAACT	+	TGG
129	CCTGTTCGTGTCAAGGGAAT	+	TGG
130	AGGCAGAGCATTCTGATAGT	+	TGG
131	TTGGGACAGATTCTATATTA	+	AGG
132	AGCGCGAGCTATTTCTTGGC	+	TGG
133	TTGGCTGGTGCTGCAATCAC	+	TGG
134	GCTGGTGCTGCAATCACTGG	+	AGG
135	TGCTGAGGTACTTGAGATGA	+	TGG
136	GGTCCACCACGTGAGCCTTA	+	TGG
137	GTTGTTGCACTCTTCGCTGA	+	TGG
138	AGTGGCTTCCTGGAGAGTAA	+	AGG
139	CCCGTGACGATCAGGGACCC	+	TGG
140	ACGCCGCGGCTGCGGCGGCG	-	CCA
141	TGGACCAGGCCGTGGCGGCG	-	CCC

142	TGGCGGCGTCGGCGGCGTTC	-	CCG
143	GCGGCGAAGGCGGAGGAGAT	-	CCG
144	ACAGGATCCTCCTCCTCTCC	-	CCA
145	CCTCTCCGCCCTCTCCGAGG	-	CCT
146	TCGGGCTCTCTGTGGAAGCA	-	CCC
147	GTTGAGAAGGATGCGAAAGA	-	CCT
148	TTGACAGCAGCCGTGACTGC	-	CCA
149	ATTGGTGACTTGGTTGTCGG	-	CCG
150	ATGTTGAAGGTGATGCCTCA	-	CCT
151	CAAGCGCGAGCTATTTCTTG	-	CCT
152	GTTTGCAGGTATAACTGTAG	-	CCA
153	GAAAGCTGTTGATGTCAACA	-	CCT
154	GATGTTGCCATGACCCTTGC	-	CCT
155	TTGTTGCACTCTTCGCTGAT	-	CCG
156	CACCGGAGAAGCTGAACATC	-	CCC
157	TCGCTGCCTGCGCCGACGTG	-	CCC
158	GCGCCGACGTGCCCGTGACG	-	CCT
159	TTGGGACAGATTCTATATTA	+	AGG
160	ATCAGGGAGATCTCCGGGGC	-	CCC

Supplemental Table 7. epegRNAs designed to induce single or double mutations using the ePE2 system.

Site*	epegRNA Sequence
T173I-P177S (+17, +6)	gcagtcacggctgctgtcaagtttcagagctatgctggaaacagcatagcaagttgaaataaggctagtccgttatcaacttgaaaaagtggcaccgagtcggtgcggaacgctggaaTtgcaatgcgaTcattgacagcagccTTAAATAATTGACGCGGTTCTATCTAGTTACGCGTTAAACCAACTAGAAA
Y248H (+16)	caaaggagcagccatcagcagtttcagagctatgctggaaacagcatagcaagttgaaataaggctagtccgttatcaacttgaaaaagtggcaccgagtcggtgctcagcagtcagCacttgagtgccttgctgatggctgcAATAAGAATTGACGCGGTTCTATCTAGTTACGCGTTAAACCAACTAGAAAGGCCGGCATGGT
L249S (+12)	caaaggagcagccatcagcagtttcagagctatgctggaaacagcatagcaagttgaaataaggctagtccgttatcaacttgaaaaagtggcaccgagtcggtgcgcagtcagtactCgagtgccttgctgatggctgcAGTAAATCTTGACGCGGTTCTATCTAGTTACGCGTTAAACCAACTAGAAAGGCCGGCATGGT
Y248H-L249S (+16, +12)	caaaggagcagccatcagcagtttcagagctatgctggaaacagcatagcaagttgaaataaggctagtccgttatcaacttgaaaaagtggcaccgagtcggtgctcagcagtcagCactCgagtgccttgctgatggctgcAGTACGACTTGACGCGGTTCTATCTAGTTACGCGTTAAACCAACTAGAAAGGCCGGCATGGT
T466I (+6)	ggccatcctgtgatcatcgtgtttcagagctatgctggaaacagcatagcaagttgaaataaggctagtccgttatcaacttgaaaaagtggcaccgagtcggtgcggcaatcgacaTctacgatgatcacaggTTAAATAATTGACGCGGTTCTATCTAGTTACGCGTTAAACCAACTAGAAAGGCCGGCATGGT
Y467C (+3)	ggccatcctgtgatcatcgtgtttcagagctatgctggaaacagcatagcaagttgaaataaggctagtccgttatcaacttgaaaaagtggcaccgagtcggtgcaatcgacacctGcgatgatcacaggTAAATATTTTGACGCGGTTCTATCTAGTTACGCGTTAAACCAACTAGAAAGGCCGGCATGGT
T466I-Y467C (+6, +3)	ggccatcctgtgatcatcgtgtttcagagctatgctggaaacagcatagcaagttgaaataaggctagtccgttatcaacttgaaaaagtggcaccgagtcggtgcggcaatcgacaTctGcgatgatcacaggTAATATATTTGACGCGGTTCTATCTAGTTACGCGTTAAACCAACTAGAAAGGCCGGCATGGT
E278K (+48)	tcctttggcccttggggatggtttcagagctatgctggaaacagcatagcaagttgaaataaggctagtccgttatcaacttgaaaaagtggcaccgagtcggtgcttAaacgtaaggaatggagattagtttgtcaatgatttcgatctccacatccccaagggccaATTCTCCGTTGACGCGGTTCTATCTAGTTACGCGTTAAACCAACTAGAAAGGCCGGCATGGT
M279T (+53)	tcctttggcccttggggatggtttcagagctatgctggaaacagcatagcaagttgaaataaggctagtccgttatcaacttgaaaaagtggcaccgagtcggtgcACtttcaacgtaaggaatggagattagtttgtcaatgatttcgatctccacatccccaagggccaATTCTTCCTTGACGCGGTTCTATCTAGTTACGCGTTAAACCAACTAGAAAGGCCGGCATGGT

E278K-M279T (+48， +53)	tcctttggcccttggggatggtttcagagctatgctggaaacagcatagcaagttgaaataaggctagtccgttatcaacttgaaaaagtggcaccgagtcggtgcACtttAaacgtaaggaatggagattagtttgtcaatgatttcgatctccacatccccaagggccaATATCTTATTGACGCGGTTCTATCTAGTTACGCGTTAAACCAACTAGAAAGGCCGGCATGGT
R471K (+1)	cacctacgatgatcacaggagtttcagagctatgctggaaacagcatagcaagttgaaataaggctagtccgttatcaacttgaaaaagtggcaccgagtcggtgcgccatggccatcTtgtgatcatcgCACTTCACTTGACGCGGTTCTATCTAGTTACGCGTTAAACCAACTAGAAAGGCCGGCATGGT
M472T (+4)	cacctacgatgatcacaggagtttcagagctatgctggaaacagcatagcaagttgaaataaggctagtccgttatcaacttgaaaaagtggcaccgagtcggtgcaaggccatggccGtcctgtgatcatcgCCAATCGATTGACGCGGTTCTATCTAGTTACGCGTTAAACCAACTAGAAAGGCCGGCATGGT
R471K-M472T (+1， +4)	cacctacgatgatcacaggagtttcagagctatgctggaaacagcatagcaagttgaaataaggctagtccgttatcaacttgaaaaagtggcaccgagtcggtgcaaggccatggccGtcTtgtgatcatcgCCCAATCATTGACGCGGTTCTATCTAGTTACGCGTTAAACCAACTAGAAAGGCCGGCATGGT
S12F (+1)	ggctgcggcggcggtgtcccgtttcagagctatgctggaaacagcatagcaagttgaaataaggctagtccgttatcaacttgaaaaagtggcaccgagtcggtgctggtccaggAacaccgccgAATAATACTTGACGCGGTTCTATCTAGTTACGCGTTAAACCAACTAGAAAGGCCGGCATGGT
S24L (+13)	ccaggccgtggcggcgtcgggtttcagagctatgctggaaacagcatagcaagttgaaataaggctagtccgttatcaacttgaaaaagtggcaccgagtcggtgcgcttccgcgacAagaacgccgccgacgccgccTAAATAAGTTGACGCGGTTCTATCTAGTTACGCGTTAAACCAACTAGAAAGGCCGGCATGGT
M39V (+1)	ccgcggcgcgcgggggatggtttcagagctatgctggaaacagcatagcaagttgaaataaggctagtccgttatcaacttgaaaaagtggcaccgagtcggtgcgcacccgcaCccccccgcTATTAACATTGACGCGGTTCTATCTAGTTACGCGTTAAACCAACTAGAAAGGCCGGCATGGT
R42W (+3)	gcgcgggggatgcgggtgcgtttcagagctatgctggaaacagcatagcaagttgaaataaggctagtccgttatcaacttgaaaaagtggcaccgagtcggtgcgcccgcacccAcacccgcatccACTTAATGTTGACGCGGTTCTATCTAGTTACGCGTTAAACCAACTAGAAAGGCCGGCATGGT
G82R (+3)	gcccatcagggagatctccggtttcagagctatgctggaaacagcatagcaagttgaaataaggctagtccgttatcaacttgaaaaagtggcaccgagtcggtgctgaaccgcccTggagatctccctACATAAACTTGACGCGGTTCTATCTAGTTACGCGTTAAACCAACTAGAAAGGCCGGCATGGT
R95K (-1)	ggagaggaggaggatcctgtgtttcagagctatgctggaaacagcatagcaagttgaaataaggctagtccgttatcaacttgaaaaagtggcaccgagtcggtgctcgctctccaacaAgatcctccAAATAATTTTGACGCGGTTCTATCTAGTTACGCGTTAAACCAACTAGAAAGGCCGGCATGGT
L102P (+3)	tccgcgtctcacctcggagagtttcagagctatgctggaaacagcatagcaagttgaaataaggctagtccgttatcaacttgaaaaagtggcaccgagtcggtgctctccg

	cccCctccgaggtgaTTTAAACTTTGACGCGGTTCTATCTAGTTACGCGTTAAACC<br>AACTAGAAAGGCCGGCATGGT
S103P<br>(+1)	tccgcgtctcacctcggagagtttcagagctatgctggaaacagcatagcaagttg<br>aaataaggctagtccgttatcaacttgaaaaagtggcaccgagtcggtgctctccg<br>ccctcCccgaggtgaTTAAATTCTTGACGCGGTTCTATCTAGTTACGCGTTAAACC<br>AACTAGAAAGGCCGGCATGGT
N111G<br>(+9, +10)	ttattagggcacaacagtgggtttcagagctatgctggaaacagcatagcaagttg<br>aaataaggctagtccgttatcaacttgaaaaagtggcaccgagtcggtgcgttcag<br>caagCCgtccaccactgttgtgccGACCACTCTTGACGCGGTTCTATCTAGTTACG<br>CGTTAAACCAACTAGAAAGGCCGGCATGGT
P199L<br>(-1)	Cgaatgagggagagaccgatgtttcagagctatgctggaaacagcatagcaagttg<br>aaataaggctagtccgttatcaacttgaaaaagtggcaccgagtcggtgcaagtca<br>ccaatcAgtctctccGGGAAAGCTTGACGCGGTTCTATCTAGTTACGCGTTAAACC<br>AACTAGAAAGGCCGGCATGGT
D213G<br>(+7)	cgggttgaaacaacttggtggtttcagagctatgctggaaacagcatagcaagttg<br>aaataaggctagtccgttatcaacttgaaaaagtggcaccgagtcggtgcaaacag<br>tcgacaCccgcaccaagttgtttcTGTAGAAATTGACGCGGTTCTATCTAGTTACG<br>CGTTAAACCAACTAGAAAGGCCGGCATGGT
D298G<br>(+7)	ggcagagcattctgatagttgtttcagagctatgctggaaacagcatagcaagttg<br>aaataaggctagtccgttatcaacttgaaaaagtggcaccgagtcggtgctataga<br>atctgCcccaactatcagaatgcAAGGGAAGTTGACGCGGTTCTATCTAGTTACGC<br>GTTAAACCAACTAGAAAGGCCGGCATGGT
R299G<br>(+9)	ggcagagcattctgatagttgtttcagagctatgctggaaacagcatagcaagttg<br>aaataaggctagtccgttatcaacttgaaaaagtggcaccgagtcggtgcatatag<br>aatcCgtcccaactatcagaatgcAAAGGGAATTGACGCGGTTCTATCTAGTTACG<br>CGTTAAACCAACTAGAAAGGCCGGCATGGT
P376L<br>(+6)	ttcccataaggctcacgtgggtttcagagctatgctggaaacagcatagcaagttg<br>aaataaggctagtccgttatcaacttgaaaaagtggcaccgagtcggtgcaccgta<br>actggtTcaccacgtgagcctCCATCCCATTGACGCGGTTCTATCTAGTTACGCGT<br>TAAACCAACTAGAAAGGCCGGCATGGT
V405I<br>(+4)	atcagcgaagagtgcaacaagtttcagagctatgctggaaacagcatagcaagttg<br>aaataaggctagtccgttatcaacttgaaaaagtggcaccgagtcggtgctgaccc<br>ttgccAttgttgcactcttcTAATTATCTTGACGCGGTTCTATCTAGTTACGCGTT<br>AAACCAACTAGAAAGGCCGGCATGGT

*, The position of the mutation to the cleavage site of ePE2 is labeled in brackets.

Supplemental sequences.

>Sdd6

ATGGTGGAAACAAGAGACAAGATCATTGCGGCAAAGAGCAGGTCTGACGCTGG
CCTCCTCGCCTTTCAGCAGGCTACTAATGGTTCCATTGATAGCAGGCCAGCGGA
GGCCATCGCCAACCTACAACGGGCCAAAACACATCTTGATGAGGCGCAGCGTTT
GGTCGCAAATTCAGACGCAGCTGTTGACAACTACATAAATGCAATACTTGGAGGG
GCGTCGGCGGCGACGGCGCAACCTTCTGCTGTGATCCCGGCATCAAAACCCTC
GCGCTTCAAGCCAATGAGAACGGACCCCGCCAAGGCCGACGAGATCCGCCCTC
ATGTCGGCAAGGATAGAGCCGTCGCTACACTCTGGGATGCTGATGGCAACAGG
GTATTAGGGCTCCACAGTGCAGATGACGACGGTCCAGCCGCCACTGCCGCGTG
GAAGCCTCCGTGGCGTGATTATGTGAGGCTGCGCCGGCATGTTGAAGCACACG
CCGCTGCGCGGATGCACCAGGATGGACACAAAACCATGGTGATGTACATCAACT
TGCCGCCGTGTAAATACTTCGACGGCTGCAAGCTGAATTTGGAGGATATTCTGCC
CAAGGGTTCCACCCTGTGGATGCATCGCGTCTTTCAAAATGGCGGCACCAAAAT
TTACCAGTTCAACGGGACTGGACGAGCTTATGTT

>Sdd7

ATGCTGGAGGCCGTCCGTGCACGGCTCATCGGCGAGGGCGGCGGTCCCGGCG
CCGTACCTGAAGGAGGGGACGGCCCGCCGGCGGTGCCGGCGGAGGAGGTGG
AGAGACTACGAGGAGAACTGCCCCCTCCGGTGGTGCCCGGAACTGGGCAGAA
AACACATGGGCGGTGGATTGGTCCTGATGGTCGTGTCCGGGCTATTGTTTCAGG
ACGCGATGAGGATGCTGCTCTCGTCCACGCCCAATTGGCGGCGAAGGGCATTC
CAGATGAACCAACAAGAAACTCTGATGTTGAGCAGAAGCTCGCCGCCCACATGG
TTGCAAATGGTATCAGGCATGTGACGTTAGTCATAAACCATAGGCCGTGCCGCGG
CTTCGACGACTCCTGTGACACGCTTGTCCCTATCATATTGCCAGAAGGCTGCAC
CCTTACCGTACACGGCCAAACTGACAAGGGGATGAGGGTGAGGGTTCGCTACA
CCGGAGGGGCAAGACCATGGTGGAGC

>OsU6 template for double sgRNA construction

GTTTCAGAGCTATGCTGGAAACAGCATAGCAAGTTGAAATAAGGCTAGTCCGTTA
TCAACTTGAAAAAGTGGCACCGAGTCGGTGCTTTTTTTTGGATCATGAACCAACG
GCCTGGCTGTATTTGGTGGTTGTGTAGGGAGATGGGGAGAAGAAAAGCCCGATT
CTCTTCGCTGTGATGGGCTGGATGCATGCGGGGGAGCGGGAGGCCCAAGTACG
TGCACGGTGAGCGGCCCACAGGGCGAGTGTGAGCGCGAGAGGCGGGAGGAA
CAGTTTAGTACCACATTGCCCAGCTAACTCGAACGCGACCAACTTATAAACCCGC
GCGCTGTCGCTTGTGTG

Blue: sgRNA scaffold and polyT terminator;

Black: OsU6 promoter;
